# Identification of SKI-II as a host-protective immunomodulator against *Staphylococcus aureus* infection

**DOI:** 10.1128/mbio.01558-26

**Published:** 2026-08-10

**Authors:** Liyang Zhang, Kai Ye, Charilaos Dellis, Lewis Oscar Felix, Narchonai Ganesan, Biswajit Mishra, Aijun Zhang, Dale J. Hamilton, Yizhi Jane Tao, Eleftherios Mylonakis

**Affiliations:** 1 Department of Medicine, Houston Methodist Hospital 23534, Houston, Texas, USA; 2 Department of BioSciences, Rice University 3990, Houston, Texas, USA; 3 Department of Medicine, Weill Cornell Medical College 12295, New York, New York, USA; Universite de Geneve, Geneva, Switzerland

**Keywords:** SKI-II, Nrf2, *Caenorhabditis elegans*, immunomodulation, *Staphylococcus aureus*

## Abstract

Antibiotic resistance threatens the effectiveness of conventional antimicrobial agents, underscoring the need for host-directed therapies that enhance immune defense. Here, we present a cross-species discovery pipeline that couples a *Caenorhabditis elegans–Staphylococcus aureus* liquid-based infection screen with mammalian mechanistic validation to identify small-molecule immunomodulators. Using six *C. elegans* innate immunity reporter strains, we identified a known small molecule, SKI-II, as a previously unrecognized host-protective compound that activates the *C. elegans* SKN-1/Nrf2 oxidative-stress pathway. In mouse RAW264.7 macrophages, SKI-II binds to the ATPase pocket of VCP (Valosin-Containing Protein), activating the PERK-dependent (Protein kinase R [PKR]-like Endoplasmic Reticulum Kinase) Nrf2 signaling regulation axis that reduces the levels of pathogenic ROS (reactive oxygen species). SKI-II treatment also promotes macrophage M1 polarization and a mitochondrial metabolic shift. This work identifies VCP as a druggable node for host-directed immunomodulation and highlights SKI-II as a prototype small molecule that boosts host tolerance to infection, thereby validating our *C. elegans*-based screening platform for discovering immunomodulators active in mammalian systems.

**IMPORTANCE:**

Enhancing host immunity is a promising strategy to combat *S. aureus* infection, particularly multidrug-resistant strains. In this study, we applied a *C. elegans* liquid-based infection screening model to identify the immunomodulatory compound SKI-II. We demonstrate that SKI-II protects against *S. aureus* infection in both nematodes and mouse macrophages by regulating host oxidative stress pathways rather than directly targeting the pathogen. We reveal a regulatory circuit in which VCP functions as a central node controlling cellular ROS responses and activating the downstream PERK-dependent Nrf2 antioxidant signaling pathway. These findings advance our understanding of host cellular responses to bacterial infection and highlight VCP as a druggable target for host-directed therapeutic strategies against infectious diseases.

## INTRODUCTION

Host-directed anti-microbial therapy is considered to be a promising strategy for combating infections that cannot be controlled with traditional antibiotics by enhancing immune defense or improving host tolerance (1–4). To accelerate the discovery of host-directed therapeutics, systematic pipelines for identifying immunomodulators remain a critical need. The nematode *Caenorhabditis elegans* offers a whole-animal platform for high-throughput discovery of immunomodulatory compounds due to its conserved immune pathways and strong genetic similarity to humans (5–7). Despite lacking an adaptive immune system, *C. elegans* possesses a conserved innate immune network that in some key aspects is similar to that of humans (8, 9). These include pattern recognition and lectin receptors (the lectin *clec-60*) (10), the antimicrobial programs, such as the p38/PMK-1 MAPK and DAF-2/DAF-16 signaling pathways (11, 12), antimicrobial peptide responses such as *nlp-29* (13), and the SKN-1/Nrf2 oxidative stress pathway that promotes host protection during infection (14). The evolutionary conservation of these immune pathways establishes *C. elegans* as a tractable and translational model for discovering immunomodulatory compounds and druggable targets that enhance host resilience to infection.

The *C. elegans* SKN-1/Nrf2 pathway is a regulatory axis that integrates redox homeostasis with innate immune defense. During infection, host-produced ROS (reactive oxygen species) results in oxidative stress and host tissue damage (15, 16). SKN-1 in *C. elegans* and its mammalian ortholog Nrf2 orchestrate antioxidant defenses by inducing genes involved in glutathione biosynthesis, xenobiotic detoxification, and proteostasis maintenance (17, 18). At a mechanistic level in mammals, ROS-mediated oxidation of KEAP1 cysteines disrupts the KEAP1–CUL3–RBX1 E3 ubiquitin ligase complex, thereby enabling Nrf2 stabilization and nuclear translocation (19). Besides, Nrf2 stability and activity are also modulated through KEAP1-independent mechanisms. For example, the AAA^+^ ATPase VCP/p97 participates in proteostasis-associated Nrf2 turnover (20). Moreover, and relevant to the data presented in this paper, the ER-stress sensor kinase PERK phosphorylates Nrf2, thereby activating Nrf2-mediated signaling in response to the unfolded protein stress (21), while more recent work suggests that PERK-mediated Nrf2 activation may also occur through ATF4-dependent transcriptional upregulation of NRF2 rather than direct phosphorylation alone (21, 22). The integration of these redox, proteostatic, and ER-stress pathways in infected cells remains incompletely understood, presenting new opportunities for mechanistic insight and therapeutic intervention.

*S. aureus* is a major Gram-positive human pathogen that causes a wide range of infections, and the emergence of methicillin-resistant *S. aureus* (MRSA) strains has further increased its clinical significance (23, 24). To expand treatment options for the highly virulent MRSA strains, high-throughput screening to identify novel compounds that inhibit *S. aureus* infection has been extensively pursued (25–28). In previous work, we screened several compound libraries containing over 82,000 unique molecules to discover candidates with antimicrobial activity against *S. aureus* in a *C. elegans—S. aureus* infection model (6). Among the hits, we found a new class of synthetic retinoid antibiotics that could effectively eliminate bacterial persisters (6), a repurposed agent that could inhibit the growth of *Helicobacter pylori* (29), and a kinase inhibitor (Bay 11-7085) that combats *S. aureus* biofilms (30). However, hits from the screening of these libraries that exhibit putative immunomodulatory effects have not yet been studied in detail.

Here, we deploy a high-throughput *C. elegans* immune-reporter screening platform to discover small-molecule immunomodulators that enhance the host immune response or enhance host tolerance to pathogen infection. Specifically, we identified the low-molecular-weight compound SKI-II, previously identified as a sphingosine kinase inhibitor, as a hit candidate that improves survival during *S. aureus* infection without direct antibacterial activity. Our studies show that SKI-II binds to VCP and activates the PERK-dependent Nrf2 signaling pathway to couple ER stress and redox defenses, suggesting a tractable route to bolster disease tolerance. SKI-II has not been previously reported to exhibit immunomodulatory activity in the context of host defense against bacterial infection although prior studies have demonstrated SKI-II-mediated Nrf2 activation and cytoprotective effects in other settings (31, 32). Together, these findings identify SKI-II as a prototype small molecule that strengthens host immunity as well as establishing a framework for discovering host-directed therapeutics against bacterial infection.

## RESULTS

### Identification of putative immunomodulatory compounds using a *C. elegans* liquid-based infection model

In previously published work from our laboratory, over 82,000 small synthetic molecules were screened using an automated liquid-based *C. elegans*—MRSA killing assay for compounds that blocked *S. aureus* MRSA strain MW2-mediated killing, leading to the identification of 185 compounds (6) (Fig. S1A). To discover compounds that potentially enhance host immunity, a total of 133 commercially available compounds (133/185, 71.9%) were screened (Fig. S1A) by monitoring GFP expression in six *C. elegans* reporter lines that contained fusions to the promoters of six immunity-related genes to GFP. Specifically, the six promoters were derived from *gst-4* (SKN-1/Nrf2 pathway), *dod-24* (DAF-2/DAF-16 insulin-like signaling pathway)*, irg-5* (p38 MAPK pathway)*, nlp-29* (antimicrobial peptide secretion), *clec-60* (pathogen pattern recognition)*,* and *irg-1* (ZIP-2-dependent pathway). GFP levels in these reporter strains were measured using a Cytation 3 multimode reader every 2 h over a 24-h period, resulting in a total of 13 time points in a liquid setting. For each compound, *z*-scores were calculated at each time point using either absolute GFP intensity or values normalized to the initial time point (*t* = 0) based on the mean and standard deviation of all compounds at the corresponding time point. A compound was defined as a hit if the *z*-score of either the absolute or normalized GFP values exceeded 2 at more than half of the time points (at least 7 out of 13 time points). From this screen, we identified five compounds corresponding to *gst-4p::gfp*, eight for *nlp-29p::gfp*, and three for *irg-1p::gfp* (Table 1). No compounds activated the *clec-60*, *dod-24*, or *irg-5* reporter strains.

**TABLE 1:** Potential immunomodulators were identified from the screen^*a*^

	Hits	Cat. 1 *z*-score	Cat. 2 *z*-score	Smiles	Predicted toxicity LD_50_ (mg/kg)	Predicted drug likeness				
						Lipinski	Ghose	Veber	Egan	Muegge
*gst-4p* *::gfp*	J10	3.70	1.52	C1=CC(=CC=C1C2=CSC(=N2)NC3=CC=C(C=C3)O)Cl	1,000	YES	YES	YES	YES	YES
	D5	4.10	0.84	CCSc1ccc(SC#N)s1	80	YES	NO	YES	YES	YES
	K6	4.03	5.49	N1(CCc(cc2)ccc2Cl)C(=O)C=CC1=O	600	YES	YES	YES	YES	YES
	I4	2.17	0.03	Nc1snc(SC#CC(F)(F)F)n1	3,000	YES	NO	YES	YES	YES
	M11	6.05	4.58	Nc1sc(Sc2sc([*N*+](=O)[O-])cn2)nn1	600	YES	NO	NO	NO	NO
*nlp-29p* *::gfp*	K8	2.37	3.61	Oc1c(ccc2cccnc12)C(N1CCOCC1)c1ccc(cc1)C(F)(F)F	200	YES	YES	YES	YES	YES
	G5	2.81	2.65	n1c(c2ccc(c(F)c2)OC)nc(nc1Nc(cc3)ccc3C)N	2,000	YES	YES	YES	YES	YES
	J8	2.02	4.31	O=C(N/N=C/c1ccc(O)c(O)c1O)c1ccc(CSc2nc3ccccc3o2)cc1	375	YES	YES	NO	NO	NO
	A10	3.37	6.88	C1=CC=C(C=C1)C2=CC(=O)C3=C(O2)C=C(C(=C3O)O)O	3,919	YES	YES	YES	YES	YES
	A9	2.26	3.68	Oc1c(ccc2cccnc12)C(N1CCCC1)c1ccc(cc1)C(F)(F)F	200	YES	YES	YES	YES	YES
	B9	0.79	4.68	O=C1C=C(NC2=CC=CC=C2)C(=O)C2=CC=CN=C12	2,000	YES	YES	YES	YES	YES
	B1	3.58	6.62	CCn1cc(C(=O)O)c(=O)c2cc(F)c(cc12)N1CCN(C)CC1	1,000	YES	YES	YES	YES	YES
	A11	2.10	2.97	CC1=C(C=CC(=C1)I)NC2=C(C(=C(C=C2C(=O)NOCC3CC3)F)F)F	800	YES	NO	YES	NO	NO
*irg-1p* *::gfp*	A8	10.83	−1.45	O=C(\C=c1/[nH]c(=O)/c(=C/c2ccsc2)\s/1)c1ccco1	1,000	YES	YES	YES	YES	YES
	A10	9.45	−0.83	C1=CC=C(C=C1)C2=CC(=O)C3=C(O2)C=C(C(=C3O)O)O	3,919	YES	YES	YES	YES	YES
	K1	5.68	0.04	COc1cc(/C=C2\SC(=O)NC/2=O)cc(Br)c1O	760	YES	YES	YES	YES	YES
	I10	4.68	0.14	Oc1c(Br)cc(\C=C(\c(c2N3)cc(I)cc2)/C3=O)cc1Br	1,000	NO	NO	YES	YES	YES

^*a*^ Cat.1 *z*-score was calculated based on the absolute GFP values. Cat.2 *z*-score was calculated based on the normalized GFP values. The average *z*-score of 13 time points is shown.

To prioritize compounds for further studies, we evaluated the drug likeness and toxicity of the hits using SwissADME (http://www.swissadme.ch) (33) and ProTox-3.0 (https://tox.charite.de/protox3/) (34), respectively (Table 1) (Fig. S1A). Based on predictions indicating high drug likeness and low toxicity, compound J10 emerged as the top hit from the *gst-4p::gfp* screen, while compound A10 was identified as the best hit from both the *nlp-29p::gfp* and *irg-1p::gfp* screens. Compound A10, known as “baicalein,” is a flavonoid extracted from the roots of *Scutellaria baicalensis* and has been reported to exhibit immune-modulating properties (35) and broad medicinal activity on *S. aureus* (36). Therefore, we decided to focus further studies on compound J10, previously referred to as SKI-II (4-[[4-(4-chlorophenyl)−2-thiazolyl]amino]phenol) (Fig. S1A) (37). It was initially identified as a sphingosine kinase inhibitor (and was named SKI-II accordingly) (38). SKI-II has not been previously reported to exhibit immunomodulatory activity or antimicrobial activity against *S. aureus*.

**Fig 1:**
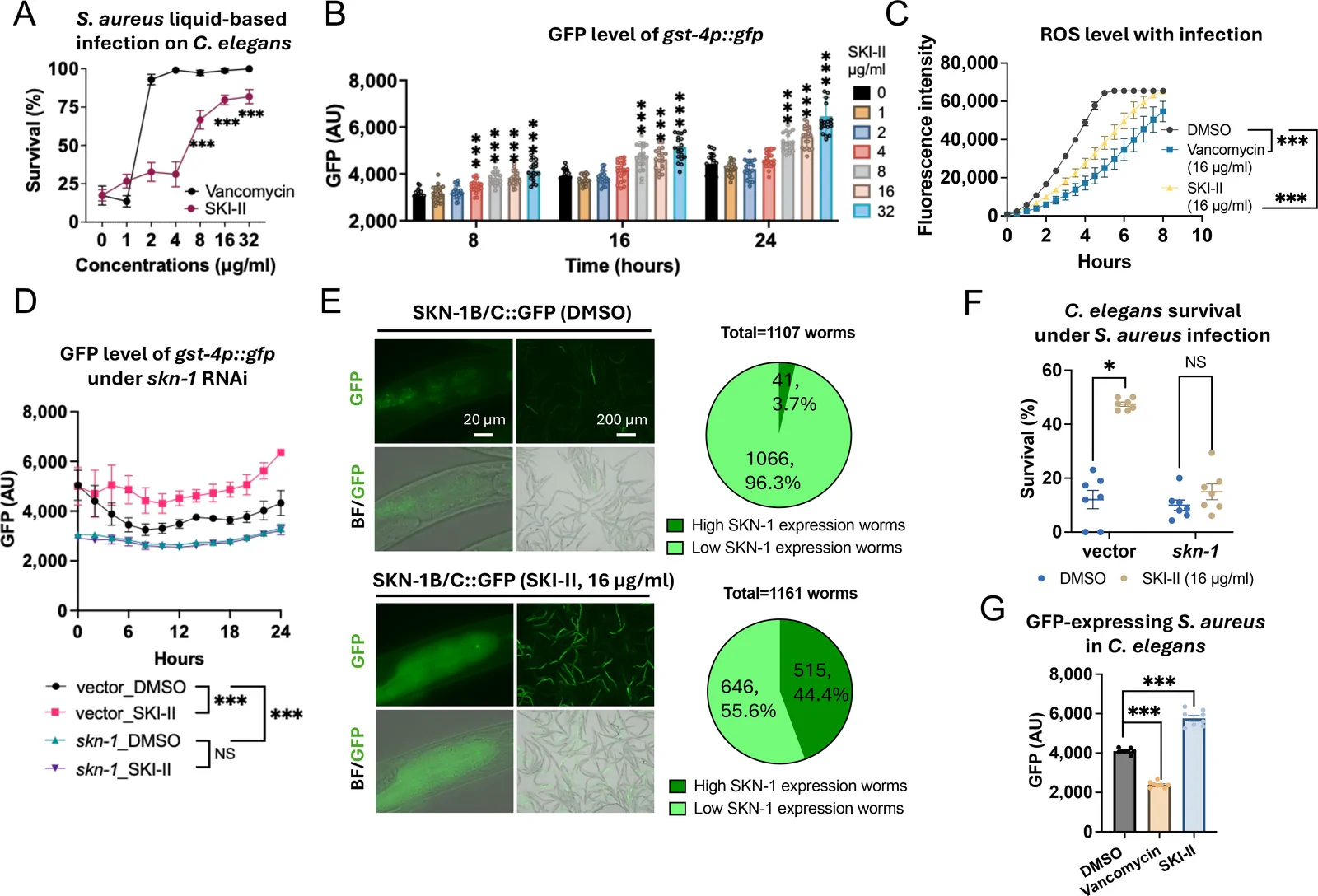
SKI-II enhances host defense in *C. elegans*. (**A**) Survival of *C. elegans* AU37 infected with *S. aureus* MW2 in liquid and treated with DMSO control or with various concentrations of SKI-II. Worm survival was calculated after 5 days of infection. One-way ANOVA followed by Dunnett’s multiple comparison test compares each SKI-II treatment to the no-treatment control at each time point (*n* = 6, number of biological replicates). ***: *P* < 0.001. (**B**) GFP intensity of *gst-4p::gfp* worms treated with SKI-II (0-32 μg/mL) for 8, 16, and 24 h. Fluorescence measured from ~50 worm pools. One-way ANOVA followed by Dunnett’s multiple comparison test compares each SKI-II treatment to the no-treatment control at each time point (*n* = 20, number of biological replicates). ***: *P* < 0.001. (**C**) ROS levels in *C. elegans* AU37 worms with *S. aureus* MW2 infection following treatment with DMSO (control), 16 μg/mL of SKI-II, or 16 μg/mL of vancomycin. The ROS level was obtained from a pool of ~50 worms. Area Under the Curve (AUC) values were calculated for each replicate, and statistical comparisons were performed using one-way ANOVA followed by Dunnett’s multiple comparison test for panel (*n* = 3, number of biological replicates). ***: *P* < 0.001. (**D**) GFP expression in *gst-4p::gfp* worms treated with DMSO or 16 μg/mL of SKI-II over time under RNAi targeting: empty vector (control) and *skn-1*. The GFP intensity corresponded to the fluorescence level from a pool of ~50 worms. AUC values were calculated for each replicate, and statistical comparisons were performed using an unpaired *t*-test between DMSO and SKI-II treatment groups (*n* = 3, number of independent experiments). NS: *P* > 0.05. ***: *P* < 0.001. (**E**) GFP levels in *C. elegans skn-1B/C::gfp* worms treated with DMSO control or 16 μg/mL of SKI-II for 24 h. Left: the representative images of single and multiple worms; right: pie charts showing the percentage of worms with high GFP expression (dark green) and low GFP expression (light green). (**F**) Survival of *C. elegans* AU37 worms subjected to RNAi (empty vector or *skn-1*), infected with *S. aureus* MW2, and treated with DMSO or SKI-II. The survival was calculated after 5 days of infection. Statistical analysis was performed using an unpaired *t*-test (*n* = 7, number of biological replicates). NS: *P* > 0.05. *: *P* < 0.05. (**G**) GFP levels in the intestines of *C. elegans* AU37 worms infected with GFP-expressing *S. aureus* and treated with DMSO, vancomycin, or SKI-II (16 μg/mL) for 3 days in liquid medium. Statistical analysis was performed using one-way ANOVA followed by Dunnett’s multiple comparison test (*n* = 8, biological replicates). ***: *P* < 0.001.

To evaluate the protective activity of SKI-II against *S. aureus* MRSA MW2 infection in *C. elegans*, we performed *C. elegans* liquid-based infection assays at various SKI-II concentrations and demonstrated that the SKI-II protective effect starts at about 8 μg/mL (Fig. 1A). The observed rescue effect was not due to antimicrobial activity, as SKI-II showed no significant activity against either free-living gram-positive bacteria, such as *S. aureus* (MW2) or gram-negative bacteria, including *Acinetobacter baumannii* (ATCC 19,606), *Pseudomonas aeruginosa* (PA14), *Enterobacter aerogenes* (EAE2625), or *Klebsiella pneumoniae* (H17185) (Table 2). Additionally, we tested the toxicity of SKI-II on *C. elegans* up to 64 µg/mL, the highest concentration that was possible to test based on the solubility of SKI-II in M9 media. No statistically significant difference in *C. elegans* survival was observed across SKI-II concentrations (one-way ANOVA, *P* = 0.059) although a modest reduction in survival was observed at higher doses (Fig. S1B). Taken together, the findings in this section suggest that SKI-II protects *C. elegans* from *S. aureus* infection by engaging host-directed mechanisms or by attenuating *S. aureus*-encoded virulence, rather than exerting direct antimicrobial effects.

**TABLE 2:** Minimal inhibitory concentrations (MICs) of SKI-II^*a*^

	*S. aureus* (MW2)	*A. baumannii* (ATCC 19606)	*P. aeruginosa* (PA14)	*E. aerogenes* (EAE2625)	*K. pneumoniae* (H17185)
SKI-II	>128	>128	>128	>128	>128
Vancomycin	1	–	–	–	–
Gentamicin	–	1	2	1	1

^*a*^ Unit: μg/mL. ”–,” not tested.

### SKI-II induces *gst-4* expression and decreases pathogenic ROS through the SKN-1/Nrf2 pathway in *C. elegans*

As described above, SKI-II was identified using the *gst-4* promoter fused to GFP as a reporter. To determine the lowest effective dose of SKI-II for activating *gst-4* expression, *gst-4p::gfp C. elegans* animals were treated with SKI-II at concentrations of 0, 1, 2, 4, 8, 16, and 32 μg/mL in a liquid setting. After 8 h of incubation, a modest but statistically significant increase in GFP fluorescence was observed at concentrations of 4 μg/mL and above (Fig. 1B). However, after 16 or 24 h of incubation, only concentrations of 8 μg/mL and above were able to sustain increased *gst-4* expression, indicating that the minimum effective concentration for sustained *gst-4* induction lies between 4 and 8 μg/mL (Fig. 1B), a concentration consistent with effective host protection (Fig. 1A). To ensure robust and sustained activation of the *gst-4* pathway, 16 μg/mL SKI-II was used in subsequent *C. elegans* assays.

GST-4 catalyzes the conjugation of glutathione (GSH) to a variety of toxic electrophilic species (39), thereby neutralizing and eliminating the harmful effects of toxic ROS. We hypothesized that infection of *C. elegans* with *S. aureus* increases ROS levels in *C. elegans* and that SKI-II treatment lowers ROS levels in a GST-4-dependent manner. We first measured ROS levels with and without SKI-II in uninfected *C. elegans* using the fluorescent molecular probe CM-H2DCFDA in a liquid medium (Fig. S1C). A slight decrease of ROS levels was observed. We next compared the ROS levels in *C. elegans* after 3 days of infection with *S. aureus* MW2 in liquid. We found a robust elevation in ROS levels following *S. aureus* infection, which was significantly reduced by vancomycin (Fig. 1C). Similar to vancomycin, SKI-II also reduced ROS levels in *S. aureus*-infected worms; however, the underlying mechanisms most likely differ, as vancomycin has the antimicrobial activity against *S. aureus* MW2, whereas SKI-II does not.

Given that SKI-II acts as a sphingosine kinase inhibitor (SPHK1) in mammals (38), we explored whether its effect on *gst-4* in *C. elegans* occurs through the *C. elegans* homolog of human SPHK1 (*sphk-1*). However, RNAi-mediated knockdown of *sphk-1* failed to suppress SKI-II-induced *gst-4* expression, suggesting that SKI-II was working through an alternative mechanism (Fig. S2A).

A body of published work has shown that *gst-4* in *C. elegans* is a downstream target of SKN-1 (40), a master transcription factor that controls oxidative homeostasis. We then showed that *skn-1* knockdown reduced *gst-4* expression relative to the vector control, confirming that SKN-1 positively regulates *gst-4* expression (Fig. 1D). Furthermore, loss of SKN-1 abolished SKI-II-induced GST-4::GFP, indicating that SKN-1 is required for SKI-II-mediated activation of *gst-4* (Fig. 1D). Nuclear localization of SKN-1 typically indicates the activation of its transcriptional function in regulating downstream genes (41). Using a *skn-1b/c::gfp* reporter strain, we observed increased GFP levels in both the cytoplasm and nucleus of intestinal cells upon treatment with 16 μg/mL SKI-II (Fig. 1E), indicating that SKI-II elevates overall SKN-1 expression rather than specifically promoting its nuclear translocation. Consistent with the conclusion that SKN-1 itself or the SKN-1 signaling pathway is a potential target of SKI-II, SKI-II failed to rescue *C. elegans* from *S. aureus* infection under conditions of RNAi-mediated knockdown of SKN-1 (Fig. 1F). Notably, even though SKI-II decreases *S. aureus*-mediated killing of *C. elegans*, SKI-II treatment resulted in increased *S. aureus* population in *C. elegans* rather than a reduction (Fig. 1G). This observation initially appeared counterintuitive. Previous studies have shown that infected worms can enter a sleep-like quiescent state as a protective response to infection, which is accompanied by reduced pharyngeal pumping and food intake (42, 43). Therefore, we measured pharyngeal pumping after 24 h of *S. aureus* infection in liquid and found that infection significantly reduced pumping activity, whereas SKI-II treatment restored pharyngeal pumping to near-normal levels (Fig. S1D). In the *S. aureus* infection group, there were 20% (14/70) worms with pumping rates lower than 30 times per min, whereas in the *S. aureus* infection with SKI-II group only 2.86% (2/70) of the worms exhibited pumping rates lower than 30/min. Importantly, SKI-II alone did not increase pharyngeal pumping in uninfected worms (Fig. S1D). Together, these results demonstrate that SKI-II modulates the SKN-1 pathway in *C. elegans* to defend against *S. aureus* infection.

### SKI-II requires CDC-48 for GST-4 activation

**Fig 2:**
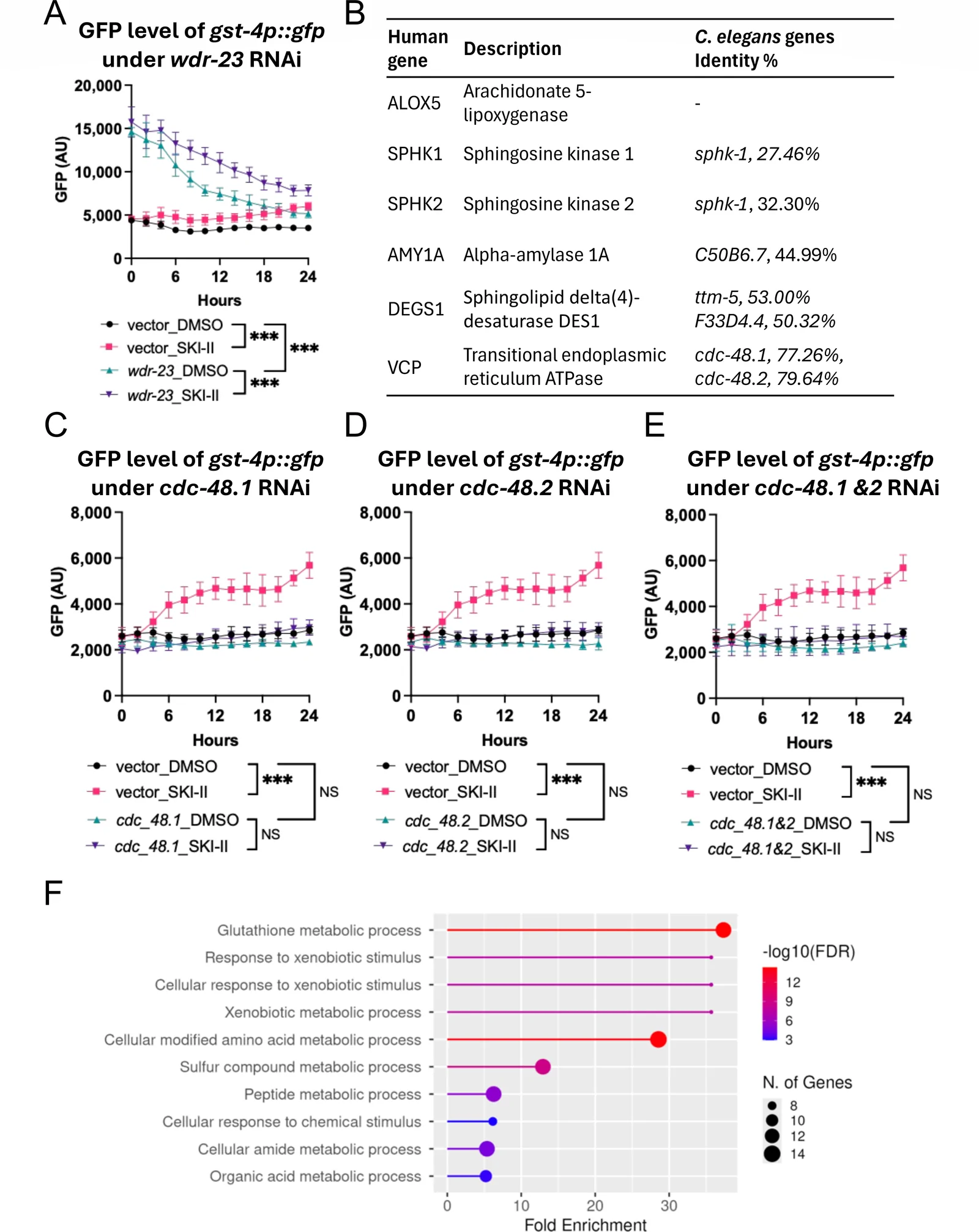
CDC-48 is required for SKI-II-mediated activation of the SKN-1/Nrf2 pathway in *C. elegans*. (**A**) GFP expression levels in *gst-4p::gfp* worms treated with DMSO (control) or SKI-II over time under empty vector or *wdr-23* RNAi. (**B**) Proteins identified as putative SKI-II interactors. The *C. elegans* genes and their levels of conservation with human genes are indicated based on the NCBI BLAST. (C–E) GFP expression in *gst-4p::gfp* worms treated with DMSO control or 16 μg/mL of SKI-II over time under RNAi targeting empty vector and (**C**) *cdc-48.1*, (**D**) *cdc-48.2*, (**E**) *cdc-48.1* and *cdc-48.2* simultaneously. (**F**) Gene Ontology (GO) Biological Process enrichment analysis comparing transcriptomic profiling of SKI-II-treated with untreated in AU37 *C. elegans*. Area under the curve (AUC) values were calculated for each replicate, and statistical significance was assessed using an unpaired *t*-test between DMSO and SKI-II treatment groups (for panels A and C–E, *n* = 3, number of independent experiments). NS: *P* > 0.05. ***: *P* < 0.001.

To further elucidate the mechanism by which SKI-II activates *gst-4* expression in *C. elegans*, we first focused on known negative regulators of SKN-1. Because small molecules typically exert their biological effects by inhibiting or modulating the function of target proteins, we reasoned that SKI-II may activate the SKN-1 pathway through the inhibition of one or more proteins that normally suppress SKN-1 activity. Therefore, we used RNAi knockdown to test whether SKI-II-mediated activation of the *gst-4p::gfp* reporter remained detectable when established SKN-1 negative regulators were depleted. Since the removal of a negative regulator is expected to increase basal SKN-1 activity and reporter expression, our analysis focused on whether SKI-II could further enhance GFP expression above this elevated baseline. WDR-23, which mediates the degradation of SKN-1 through the CUL-4/DDB-1 E3 ubiquitin system, is a principal negative regulator of SKN-1 (41). RNAi knockdown of *wdr-23* resulted in robust GFP expression on the *gst-4p::gfp* reporter compared to the vector control (Fig. 2A). However, SKI-II treatment further increased GFP expression in the *wdr-23* RNAi background, indicating an additive effect beyond the activation caused by loss of WDR-23 alone (Fig. 2A). We then used RNAi to knock down several additional known SKN-1 negative regulators, including *gsk-3, sgk-1, akt-1, akt-2,* and double *akt-1/atk-2*. In all cases, SKI-II retained its ability to induce additive *gst-4* expression (Fig. S2B through F). These results suggest that SKI-II-mediated activation of SKN-1 is not absolutely dependent on any of these individual negative regulatory pathways.

Because the knockdown of several *skn-1* negative regulators failed to identify a potential target for SKI-II, we sought to identify potential SKI-II-interacting proteins using BindingDB, which is a publicly accessible database of experimentally determined binding affinities (https://www.bindingdb.org/rwd/bind/index.jsp). Six human proteins known to interact with SKI-II were identified, including ALOX5 (44), AYM1A (45), DEGS1 (46), SPHK1 (47), SPHK2 (48), and VCP (49) (Fig. 2B). Based on BLAST analysis (https://blast.ncbi.nlm.nih.gov/Blast.cgi), all of these proteins have homologs in *C. elegans* except ALOX5. We knocked down each homolog gene in the *gst-4p::gfp* reporter strain using RNAi and found that loss of *cdc-48.1* or *cdc-48.2* (VCP homologs), but not of the other homologs, abolished SKI-II-induced *gst-4* expression, indicating that CDC-48/VCP was required for SKI-II activating the SKN-1/Nrf2 pathway (Fig. 2C through E; Fig. S2G through I). Notably, RNAi knockdown of CDC-48 did not alter GFP expression in the *gst-4p::gfp* reporter strain (Fig. 2C through E) or affect SKN-1 expression and nuclear localization (Fig. S3A), suggesting that CDC-48 is required for SKI-II-induced SKN-1 activation rather than acting as either a positive or negative regulator of basal SKN-1 activity. To investigate whether SKI-II inhibits or enhances CDC-48 function, we employed two ER stress assays, as a primary function of CDC-48 is the maintenance of ER homeostasis. Previous studies have shown that loss of CDC-48 induces ER stress, as indicated by the activation of the *hsp-4p::gfp* reporter, a well-established marker of the unfolded protein response (50). Treatment with dithiothreitol (DTT), a reducing agent that breaks disulfide bonds and thereby disrupts oxidative protein folding in the ER (51), induced ER stress in the *hsp-4p::gfp* reporter strain (Fig. S3B). However, SKI-II alone did not induce ER stress, nor did it exert an additive effect in DTT-treated worms (Fig. S3B). Furthermore, SKI-II failed to accelerate recovery from ER stress following DTT pretreatment (Fig. S3C). Together, these results suggest that SKI-II neither inhibits nor enhances CDC-48 function under the conditions tested.

We next explored the mechanism by which SKI-II drives SKN-1 activation in *C. elegans* AU37 worms by comparing the transcriptional profiles with and without SKI-II treatment. Gene Ontology (GO) Biological Process enrichment analysis using ShinyGO (https://bioinformatics.sdstate.edu/go74/) revealed that the glutathione metabolic process was the most significantly enriched pathway, including the genes *gst-4, gst-5, gst-7, gst-8, gst-10, gst-12, gst-14, gst-16, gst-24, gst-30, gst-31, gst-35,* and *gst-37* (Fig. 2F). These results further support that the *gst-4p::gfp* reporter accurately reflects endogenous *gst-4* transcriptional activation following SKI-II treatment. In addition, SKI-II induced a strong detoxification and xenobiotic stress-response transcriptional program in *C. elegans* (Fig. 2F), which is highly consistent with activation of the SKN-1/Nrf2 pathway. Whether CDC-48 is involved in SKN-1 activation or xenobiotic stress is further discussed in the Discussion section.

### SKI-II activates the Nrf2-mediated oxidative stress response in macrophages

Having established the immunomodulatory effects of SKI-II in *C. elegans*, we next investigated whether these activities of SKI-II are conserved in mammalian systems. We first assessed the potential toxicity of SKI-II using a cell viability assay. SKI-II showed significant cytotoxicity at over 32 μg/mL in the mouse macrophage cell line RAW264.7 (Fig. S4A). In contrast, SKI-II caused no hemolysis of human red blood cells up to 64 μg/mL (Fig. S4B). Higher concentrations of SKI-II could not be tested due to solubility limitations. We chose a concentration of 4 μg/mL, which does not cause detectable cytotoxicity in RAW264.7 macrophages, to map the effect of SKI-II on the global immune network by comparing the transcriptional profiles of RAW264.7 macrophages treated with SKI-II and a DMSO control.

**Fig 3:**
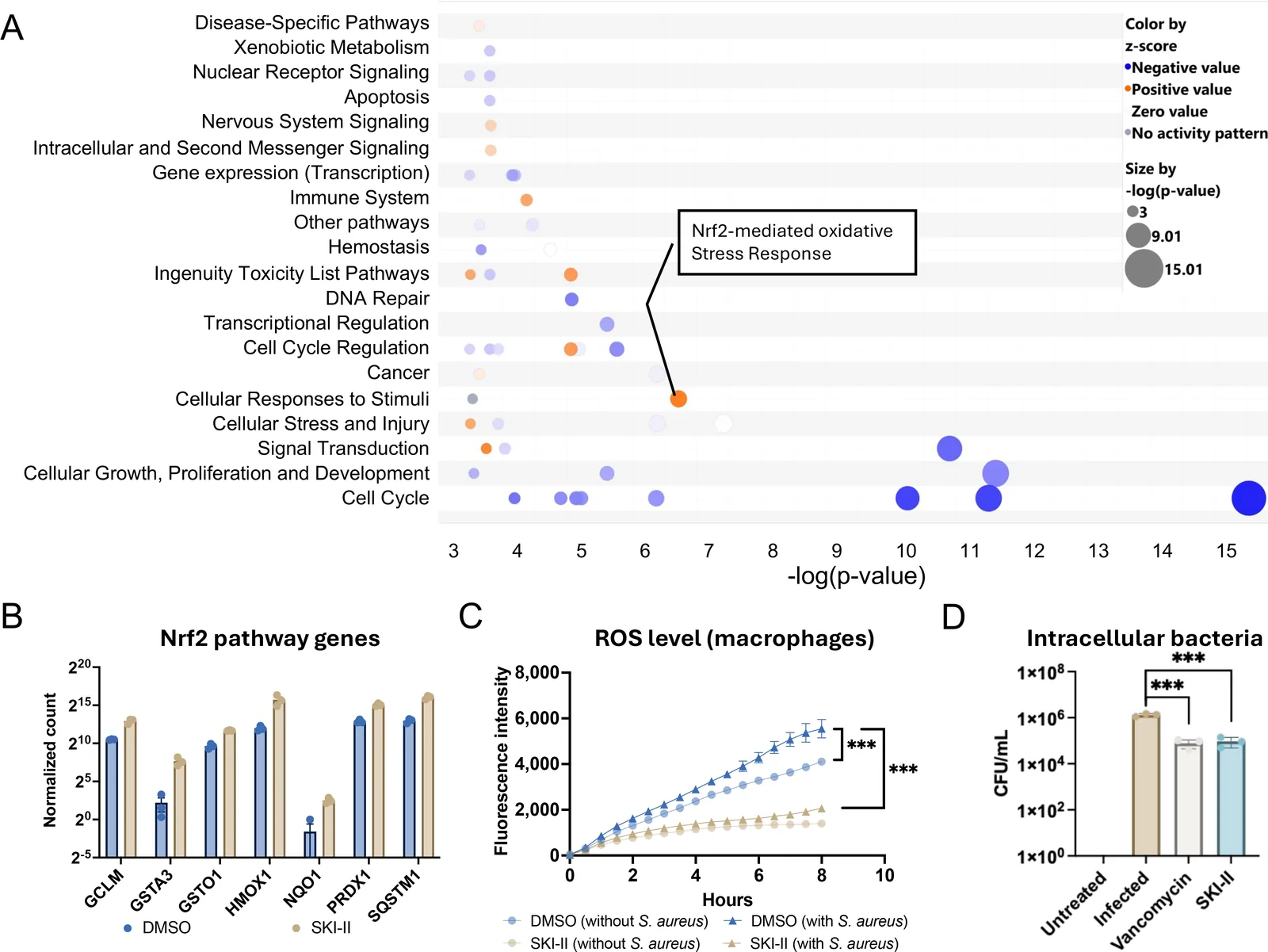
SKI-II activates the mammalian Nrf2 pathway to mitigate infection-induced ROS. (**A**) Ingenuity Pathway Analysis (IPA) of the transcriptional profile comparing 4 μg/mL of SKI-II-treated cells to DMSO control in murine RAW264.7 macrophage cells. The transcriptional profiles were obtained after 24 h of incubation. Blue indicates downregulation, while orange indicates upregulation. The bubble size represents the degree of gene overlap with a given pathway. (**B**) Upregulation of Nrf2 pathway-associated genes following 4 μg/mL of SKI-II treatment compared to the DMSO control. (**C**) Intracellular ROS levels in RAW264.7 macrophages, with or without *S. aureus* MW2 infection with an MOI of 10, treated with DMSO or 4 μg/mL of SKI-II. Area under the curve (AUC) values were calculated for each replicate, and statistical significance was assessed using an unpaired *t*-test between DMSO and SKI-II treatment groups. (*n* = 3, number of biological replicates). ***: *P* < 0.001. (**D**) Intracellular *S. aureus* MW2 in RAW264.7 macrophages with an MOI of 10, treated with DMSO (infected), 4 μg/ml of vancomycin, or 4 μg/mL of SKI-II. Statistical analysis was performed using one-way ANOVA followed by Dunnett’s multiple comparison test. (*n* = 3, number of biological replicates). ***: *P* < 0.001.

We conducted pathway analysis on genes with a log2 fold change of ≤−2 (downregulated) or ≥2 (upregulated) in Ingenuity Pathway Analysis (IPA; Qiagen). In total, 212 downregulated and 275 upregulated genes were included in this pathway analysis. We found that the most significantly upregulated pathway was the Nrf2-mediated oxidative stress response (Fig. 3A), consistent with our observations in *C. elegans*, where SKI-II regulates the SKN-1 pathway, the ortholog of Nrf2. Key downstream target genes of the Nrf2 pathway, including GCLM, GSTA3, GSTO1, HMOX1, NQO1, PRDX1, and SQSTM1, were markedly induced upon SKI-II treatment of RAW264.7 macrophages (Fig. 3B).

To further explore oxidative stress modulation by SKI-II in mouse macrophages, we assessed ROS levels in the presence or absence of *S. aureus* infection of RAW 264.7 cells. ROS levels significantly increased following infection (Fig. 3C); however, SKI-II treatment consistently suppressed ROS accumulation to minimal levels (Fig. 3C). These findings indicate that key SKI-II-mediated antioxidant effects are conserved between *C. elegans* and mammalian cells. We next assessed intracellular *S. aureus* after SKI-II treatment and observed a significant reduction in bacterial burden, suggesting that SKI-II exerts a protective effect by enhancing host defense in macrophages (Fig. 3D). Taken together, these data show that SKI-II activates Nrf2 signaling and confers both cytoprotective effects and improved antimicrobial function against *S. aureus* infection.

### SKI-II inhibits VCP by binding to the VCP ATPase domain

**Fig 4:**
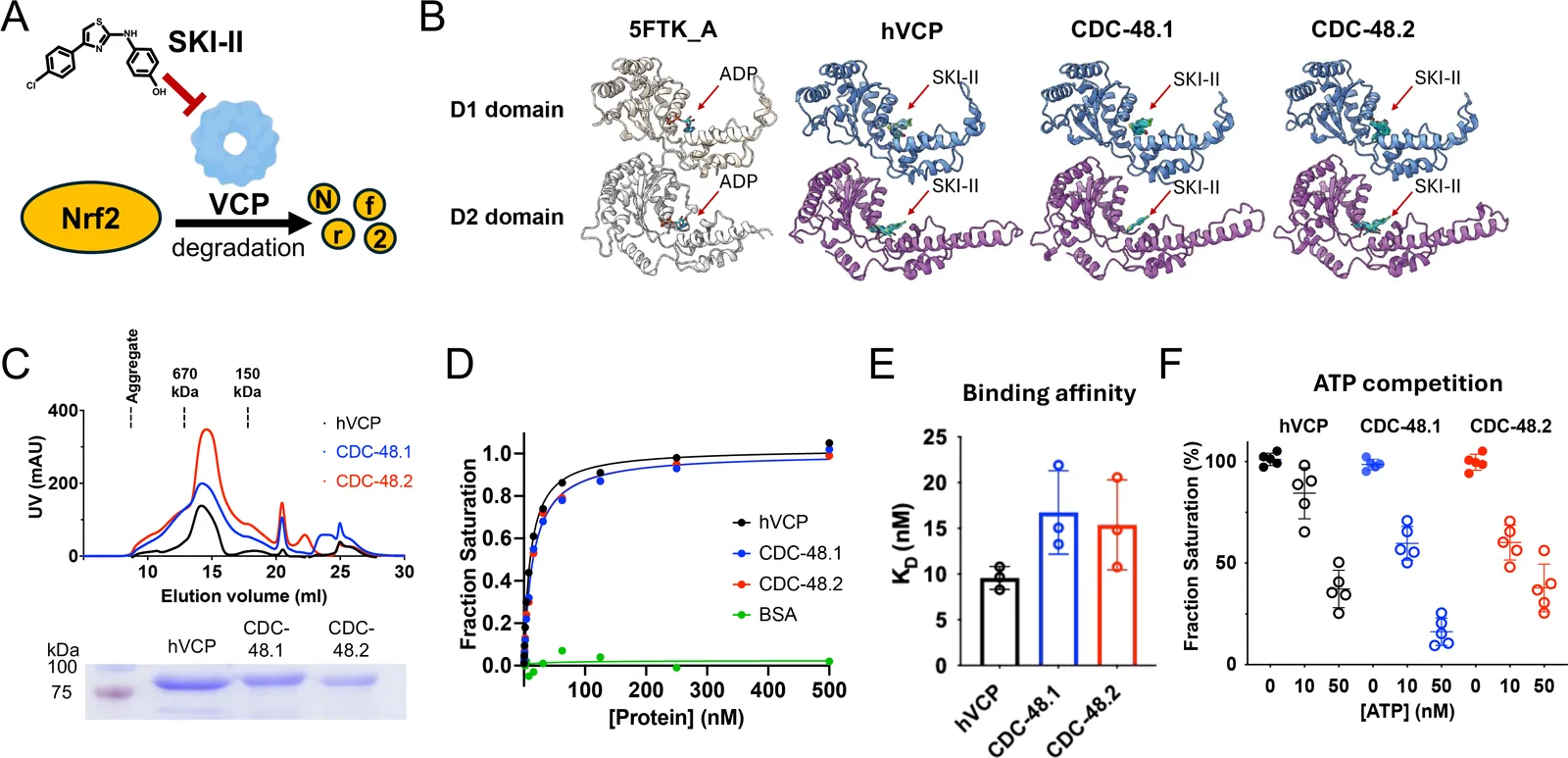
SKI-II binds to the ATPase domain on VCP/CDC-48. (**A**) Diagram of VCP-mediated Nrf2 degradation. SKI-II inhibits VCP ATPase activity, thereby reducing Nrf2 degradation and stabilizing Nrf2. (**B**) Docking of SKI-II on human VCP and *C. elegans* CDC-48.1 and CDC-48.2 D1 and D2 ATPase domains. Human VCP (PDB 5FTK, chain A) is a cryo-EM structure, and CDC-48.1/CDC-48.2 are predicted by AlphaFold3. (**C**) Size exclusion analysis (the upper panel) of human VCP, *C. elegans* CDC-48.1, and CDC-48.2 recombinantly expressed in *E. coli*. All three homologs appear to form homohexamers. SDS-PAGE analysis (the lower panel) of the expression and purification of the monomeric hVCP, CDC-48.1, and CDC-48.2. (**D, E**) Fluorescence polarization analysis of SKI-II binding to human VCP or *C. elegans* CDC-48.1. BSA served as the negative control. (**F**) Effect of ATP on the displacement of SKI-II from human VCP, *C. elegans* CDC-48.1, and CDC-48.2 can be observed in FP assays. The FP measurements showed that this displacement effect is ATP-concentration dependent. Proteins at 500 nM concentration were preincubated with 10 nM SKI-II before ATP titration.

Next, we investigated how SKI-II regulates Nrf2 in mammalian cells. Previous studies have shown that VCP (the homolog of CDC-48 in *C. elegans*) extracts ubiquitinated Nrf2 from the KEAP1–CUL3 E3 ligase complex, thereby promoting its proteasomal degradation (20) (Fig. 4A). In addition, *in vitro* biochemical assays have demonstrated that SKI-II inhibits VCP ATPase activity (49). Therefore, we reasoned that SKI-II may modulate Nrf2 through VCP (Fig. 4A).

However, previous studies did not determine the exact mechanism of how SKI-II inhibits the ATP hydrolysis activity of VCP. Here, we performed unbiased molecular docking of SKI-II to monomeric VCP and VCP homologs CDC-48.1 and CDC-48.2 using DiffDock (52). The monomeric unit of human VCP used in docking was extracted from a published PDB structure (PDB ID: 5FTK), while the monomeric unit of *C. elegans* CDC-48.1 and CDC-48.2 (VCP homologs) was predicted with AlphaFold3 (53). Across both the D1 and D2 ATPase domains of VCP and the two CDC-48 variants, SKI-II was predominantly located in the nucleotide-binding pocket present in the ADP-bound cryo-EM structure (Fig. 4B). These docking experiments also showed that SKI-II did not bind to the previously identified allosteric surface pockets (54). With these docking results, we reasoned that SKI-II interacts with the D1/D2 ATPase active sites and exerts competitive inhibition on ATPase activity (Fig. 4B).

To test the hypothesis that SKI-II is a competitive inhibitor of ATP binding to VCP and the CDC-48 homologs, these proteins were recombinantly expressed in *E. coli*. All three proteins, VCP, CDC-48.1, and CDC-48.2, were purified as presumptive homohexamers of ~90 kDa monomeric units (Fig. 4C). We next performed fluorescence polarization (FP) assays using SKI-II and recombinantly purified His-VCP, or *C. elegans* CDC-48.1, and CDC-48.2 (Fig. S5). Nonlinear regression of binding isotherms indicated that SKI-II binds to all three proteins with dissociation constants of 9.6 ± 1.3 nM for human VCP, 16.7 ± 4.6 nM for nematode CDC-48.1, and 15.4 ± 4.9 nM for CDC-48.2 (Fig. 4D and E). Similar SKI-II FP maximum values were observed in VCP, CDC-48.1, and CDC-48.2, indicating the consistency of the FP assays.

To further determine whether SKI-II inhibits VCP’s ATPase activity through competitive inhibition, FP assays were performed with additional ATP molecules in the reaction. As shown in Fig. 4F, the presence of ATP in the FP assays significantly reduced the SKI-II FP signal, suggesting that ATP competitively displaces SKI-II from the ATPase binding sites of VCP, CDC-48.1, or CDC-48.2. This reduction in SKI-II’s FP values was ATP concentration-dependent (Fig. 4F), consistent with the hypothesis that SKI-II binds to the VCP ATPase active site and inhibits its ATPase activity through a competitive mechanism.

### SKI-II regulates Nrf2 signaling through the VCP-mediated PERK-dependent ER stress pathway

**Fig 5:**
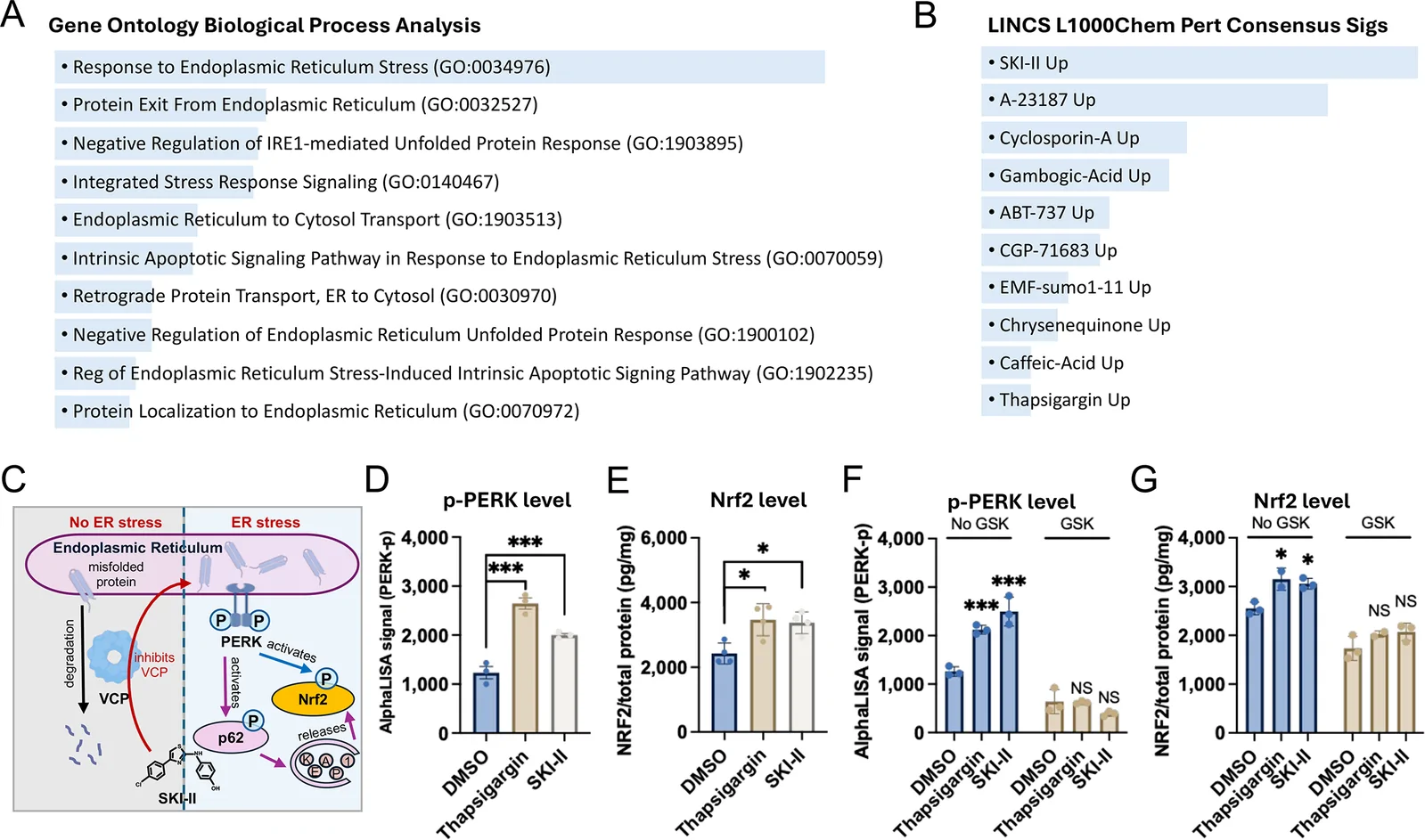
SKI-II activates Nrf2 via the VCP–PERK pathway. (**A**) Gene Ontology Biological Process analysis of significantly upregulated genes following SKI-II (4 μg/mL) treatment compared to the DMSO control. (**B**) List of compounds with LINCS L1000 ChemPert consensus signatures that significantly align with SKI-II’s upregulated gene signature. (**C**) Diagram of SKI-II–induced Nrf2 activation mediated by the PERK-dependent ER stress pathway. VCP mediates the degradation of misfold protein from the ER under normal condition (left part); SKI-II binds to and inhibits VCP, leading to ER stress and subsequent activation of PERK signaling (right part). PERK then may phosphorylate Nrf2 to promote its activation, or alternatively phosphorylate p62, which mediates KEAP1 degradation and facilitates Nrf2 nuclear entry for downstream gene regulation. (**D**) PERK phosphorylation levels under DMSO control, 0.3 μg/mL thapsigargin (positive control), and 4 μg/mL SKI-II treatments after 4 h of incubation. The phosphorylation levels were measured using an AlphaLISA assay and normalized to total protein for each group. (**E**) Nrf2 levels under DMSO control, 0.3 μg/mL thapsigargin, and 4 μg/mL SKI-II treatments after 16 h of incubation. Nrf2 was quantified by ELISA and normalized to total protein (pg/mg) for each group. (**F**) PERK phosphorylation levels without (blue) or with (yellow) PERK inhibitor GSK2606414 under DMSO control, 0.3 μg/mL thapsigargin (positive control), and 4 μg/mL SKI-II treatments after 4 h of incubation. (**G**) Nrf2 levels without (blue) or with (yellow) PERK inhibitor GSK2606414 under DMSO control, 0.3 μg/mL thapsigargin (positive control), and 4 μg/mL SKI-II treatments after 16 h of incubation. Statistical analysis was performed using one-way ANOVA followed by Dunnett’s multiple comparison test. (for panels D–G, *n* = 3, number of biological replicates). NS: *P* > 0.05. *: *P* < 0.05. **: *P* < 0.01. ***: *P* < 0.001.

Protein VCP has additional connections to the Nrf2 signaling pathway beyond its role in mediating Nrf2 degradation (20). One of the primary functions of VCP is to extract misfolded or unfolded proteins from the ER membrane and deliver them to the proteasome for degradation, a process essential for alleviating ER stress (55). Consistent with VCP inhibition by SKI-II, analysis of the significantly upregulated genes from the macrophage RAW264.7 RNA-seq data described above using Enrichr (https://maayanlab.cloud/Enrichr/) (56), revealed that SKI-II treatment strongly activates ER stress responses (Fig. 5A). Specifically, Gene Ontology (GO) Biological Process 2025 analysis indicated significant impairment of protein trafficking from the ER to the cytoplasm as well as the degradation of unfolded proteins (Fig. 5A).

In support of these conclusions, we found that the set of SKI-II upregulated genes identified in our RNA-seq studies most closely matches the set of genes upregulated by SKI-II in the LINCS L1000 Chemical Perturbation Consensus Signatures (Chem Pert Consensus Sigs) database, which compiles transcriptional profiles of cells exposed to thousands of small molecules (Fig. 5B). This match provides independent validation that our RNA-seq data faithfully captures the transcriptional signature of SKI-II treatment. Notably, several compounds that elicit upregulated gene profiles similar to SKI-II are known activators of Nrf2 signaling, such as gambogic acid (57) and caddeic acid (58) (Fig. 5B), and several ER stress inducers, including thapsigargin (59), A-23187 (60), and cyclosporin A (61) (Fig. 5B), indicating a role for SKI-II in ER stress and antioxidant responses.

The PERK–eIF2α–ATF4 pathway, the ATF6-mediated pathway, and the IRE1–XBP1 pathway are three well-characterized unfolded protein response (UPR) pathways activated in response to ER stress (62). Among these three UPR branches, the PERK pathway is most closely linked to Nrf2 signaling (63). Activated PERK (PERK-p) has been shown to directly phosphorylate Nrf2 and p62/SQSTM1, which facilitates autophagic degradation of KEAP1, thereby promoting Nrf2 activation (21, 64) (Fig. 5C). In addition, the downstream PERK effector ATF4 can induce p62/SQSTM1 expression, which was significantly upregulated following SKI-II treatment (65). To determine whether SKI-II induces PERK activation in macrophages, we measured PERK phosphorylation using an AlphaLISA assay. Thapsigargin, a specific inhibitor of the endoplasmic reticulum Ca²^+^-ATPase (SERCA) pump, was included as a positive control for ER stress. After 4 h of treatment, both thapsigargin and SKI-II significantly increased PERK phosphorylation compared with the DMSO controls (Fig. 5D). Meanwhile, we observed that both thapsigargin and SKI-II elevate cellular Nrf2 levels (16 h of treatment) (Fig. 5E). To verify whether this effect was PERK-dependent, we employed GSK2606414, a selective PERK phosphorylation inhibitor (Fig. 5F). Treatment with GSK2606414 abolished the thapsigargin- or SKI-II-induced increase in Nrf2 levels (Fig. 5G). Taken together, these findings suggest that SKI-II engages a PERK-dependent Nrf2 signaling to drive the antioxidant response.

### SKI-II drives M1 polarization of macrophages and shifts mitochondrial respiration

**Fig 6:**
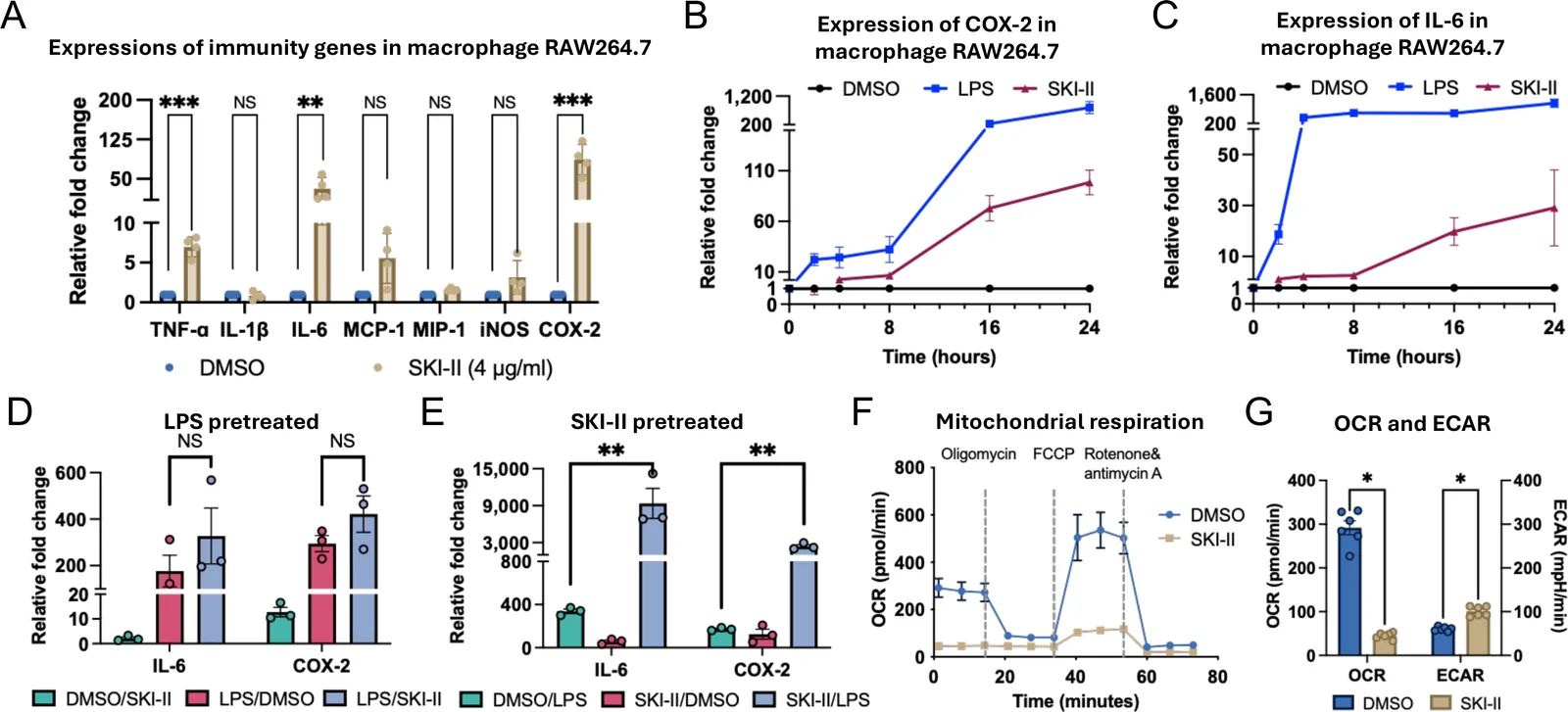
SKI-II promotes M1 macrophage polarization. (**A**) qPCR analysis of pro-inflammatory gene expression (TNF-α, IL-1β, IL-6, MCP-1, MIP-1, iNOS, and COX-2) in macrophages treated with DMSO control or 4 μg/mL of SKI-II after 24 h of incubation. Statistical analysis was performed using multiple unpaired t tests. (*n* = 4, number of independent experiments). NS: *P* > 0.05; **: *P* < 0.01; ***: *P* < 0.001. (**B and C**) Time-course expression of (**B**) COX-2 and (**C**) IL-6 in response to DMSO control, 4 μg/mL of SKI-II, or 4 μg/mL of LPS treatment. (**D**) COX-2 and IL-6 expression in macrophages treated with DMSO control or 4 μg/mL of SKI-II following 4 μg/mL of LPS pre-treatment for 16 h. (**E**) COX-2 and IL-6 expression after 4 μg/mL of LPS stimulation for 8 h, with prior 16 h of exposure to either DMSO control or 4 μg/mL SKI-II. Statistical analysis was performed using one-way ANOVA followed by Tukey’s multiple comparison test (for panels D and E, *n* = 3, number of independent experiments). NS: *P* > 0.05; **: *P* < 0.01; ***: *P* < 0.001. (**F**) Mitochondrial respiration in cells treated with DMSO control or 4 μg/mL of SKI-II. Oligomycin (1.5 µM) was used to inhibit ATP synthase (complex V) and measure ATP-linked respiration. FCCP (1 µM) was used to uncouple mitochondrial respiration and determine maximal respiration and spare respiratory capacity. Rotenone (0.5 µM) and antimycin A (0.5 µM) were used to inhibit complex I and III, respectively. (**G**) Oxygen consumption rate (OCR) and extracellular acidification rate (ECAR) under DMSO control or 4 μg/mL of SKI-II treatment. An unpaired *t*-test was performed between DMSO controls and SKI-II treatment groups for panel G. *: *P* < 0.05.

The activation of the PERK signaling branch of the unfolded protein response has been shown to induce M1-like polarization, thereby promoting the expression of inflammatory mediators (66, 67). Moreover, as shown in Fig. 3D, the enhanced antimicrobial activity following SKI-II treatment may be mediated by macrophage pro-inflammatory responses. To test the hypothesis that SKI-II drives macrophage M1 polarization, we mapped the expression of M1 marker genes, including three cytokines (TNF-ɑ, IL-1β, IL-6), two chemokines (MCP-1, MIP-1ɑ), and two inflammatory enzymes (iNOS, COX-2) in murine macrophage RAW 264.7 cells. After 24 h of SKI-II treatment, TNF-ɑ, IL-6, and COX-2 exhibited significant upregulation compared to the DMSO control (Fig. 6A). Specifically, IL-6 and COX-2 showed the most pronounced induction, with fold changes of 31 and 71, respectively (Fig. 6A). A subsequent time-course analysis indicated that elevated expression of IL-6 and COX-2 was first noticeable at 8 h post-SKI-II treatment and remained significantly elevated through 24 h (Fig. 6B and C; Fig. S6A through E).

Furthermore, we explored whether SKI-II could modulate macrophage responsiveness to a secondary inflammatory stimulus. Interestingly, SKI-II treatment alone did not significantly induce IL-6 and COX-2 expression in macrophages pre-stimulated with lipopolysaccharide (LPS), a standardized and reproducible stimulus for M1 macrophage activation. However, when cells were pre-treated with SKI-II prior to LPS exposure, there was a marked enhancement in the expression of IL-6 and COX-2, increasing by 13.8- and 27.8-fold, respectively, compared to LPS stimulation alone (Fig. 6D and E). This observation suggests that SKI-II treatment primes macrophages, enhancing their sensitivity to subsequent inflammatory signals, indicating that SKI-II sensitizes cells to subsequent inflammatory signaling. Together, our findings provide evidence that SKI-II promotes macrophage polarization toward an M1 pro-inflammatory phenotype.

As mitochondria represent one of the primary cellular sources of ROS, we investigated the impact of SKI-II on mitochondrial function using the Seahorse mitochondrial stress assay (68). After a 2-h SKI-II exposure, mitochondrial respiration was substantially reduced (Fig. 6F). Specifically, SKI-II treatment resulted in a decreased oxygen consumption rate (OCR) and simultaneously increased the extracellular acidification rate (ECAR) (Fig. 6G), reflecting a metabolic shift from mitochondrial oxidative phosphorylation to glycolysis. This metabolic reprogramming may result from multiple factors, including the increased energy demands associated with M1-like macrophage activation (69) or impaired mitochondrial function caused by disruption of mitochondrial homeostasis following VCP inhibition by SKI-II (70). Collectively, these results demonstrate that SKI-II affects the mitochondrial metabolic shift.

## DISCUSSION

The rapid emergence of antimicrobial resistance has increased interest in host-directed therapies that enhance host resilience to infection while minimizing selective pressure on pathogens. In this study, using a high-throughput *C. elegans-S. aureus* killing assay coupled with a set of *C. elegans* immune reporter strains, we reported that the small molecule SKI-II functions as a novel immunomodulator that protects *C. elegans* from *S. aureus* infection. Mechanistically, we found that SKI-II activates a VCP-associated, PERK-dependent Nrf2 response in mammalian cells, thereby promoting redox homeostasis and enhancing host tolerance to infection. SKI-II also drives M1 polarization in murine macrophages and suppresses mitochondrial respiration, thereby potentially enhancing antimicrobial ability and decreasing ROS production. Together, these findings establish SKI-II as a novel regulator of innate immunity across species, with potential therapeutic implications.

While our study is not the first to utilize *C. elegans* reporter strains to identify immunomodulatory compounds, it builds upon and addresses key limitations of prior approaches. For example, Yun et al. screened for compounds that activate the conserved p38/MAPK pathway using a reporter strain that expresses GFP under the control of the p38/MAPK reporter gene F35E12.5a (71). However, directly targeting innate immune pathways does not necessarily guarantee improved host survival during infection. Our screening pipeline began with compounds that conferred protection to *C. elegans* during an *S. aureus* infection in a large-scale *in vivo* screen, thereby prioritizing functionally relevant immunomodulators with demonstrated protective effects.

**Fig 7:**
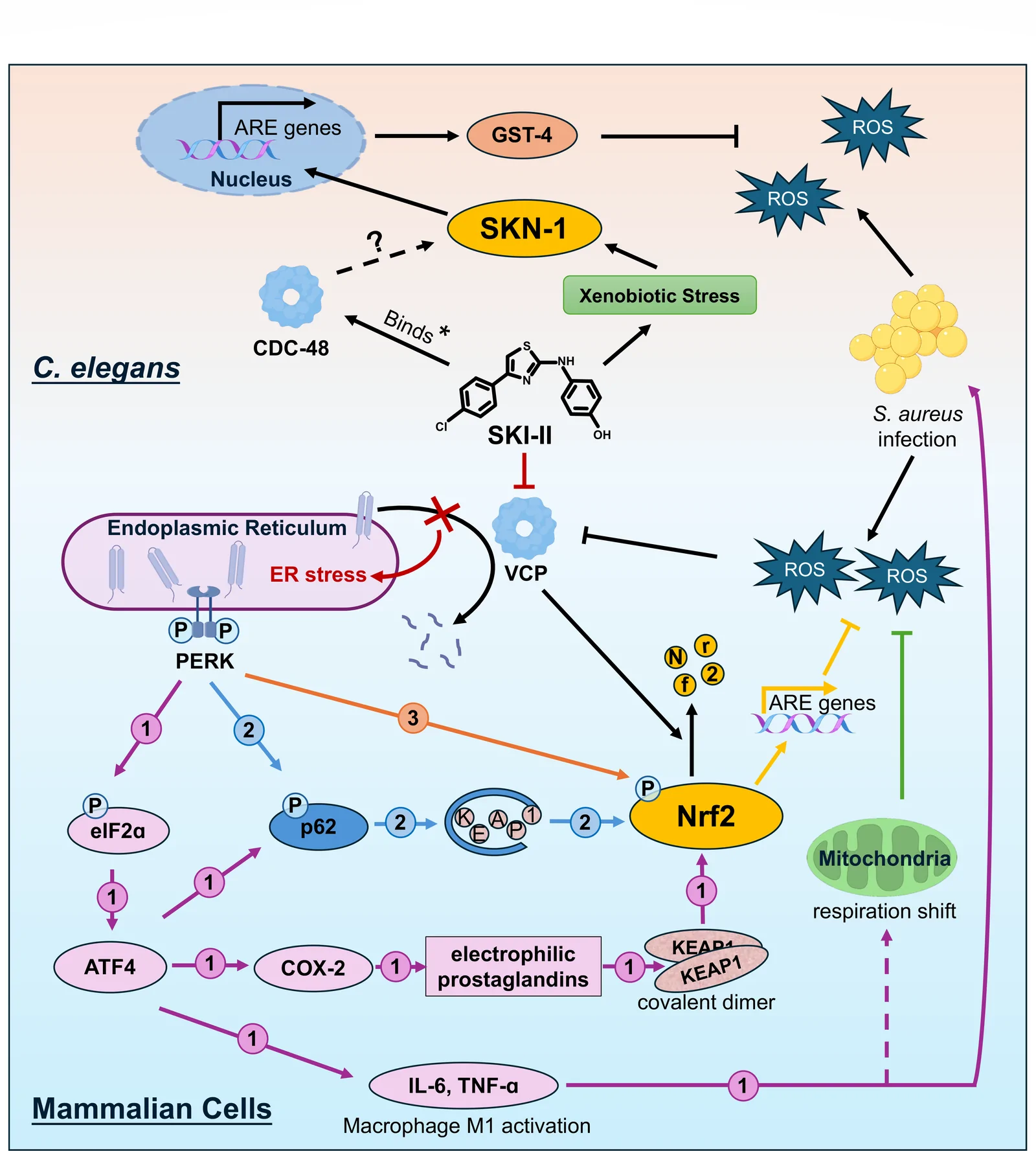
Proposed model of SKI-II-mediated activation of SKN-1/Nrf2 signaling in *C. elegans* and mammalian macrophages. The upper part shows the proposed mechanism in *C. elegans*, where SKI-II binds to CDC-48 (VCP in mammals) and induces xenobiotic stress. Furthermore, SKI-II treatment leads to SKN-1 activation. Activated SKN-1 induces antioxidant response element genes that reduce pathogenic ROS and enhance resistance to *S. aureus* infection. Unlike in the mammalian system where VCP negatively regulates Nrf2, CDC-48 in *C. elegans* does not appear to function as either a positive or negative regulator of basal SKN-1 activity; rather, CDC-48 is required for SKI-II-induced SKN-1 activation through a mechanism that remains to be determined. The connections among CDC-48, SKN-1, xenobiotic stress, and SKI-II are unknown. The lower part depicts the proposed mechanism in mammalian macrophages. SKI-II inhibits VCP, reducing Nrf2 degradation and inducing ER stress, which activates PERK signaling (red). PERK may regulate Nrf2 through three mechanisms (1, pink; 2, blue; 3, orange pathways), which are discussed but were not experimentally verified in this study. (1) Activated PERK phosphorylates eIF2α, leading to selective translation of ATF4. ATF4 promotes p62 expression, facilitating KEAP1 degradation and thereby releasing Nrf2 from KEAP1-mediated repression. In addition, ATF4 upregulates COX-2, promoting the production of electrophilic prostaglandins that induce covalent KEAP1 dimerization, further releasing Nrf2 for activation (pink pathways). (2) Activated PERK can also phosphorylate p62, enhancing its interaction with KEAP1 and promoting KEAP1 degradation, resulting in Nrf2 activation (blue pathway). (3) PERK may directly phosphorylate Nrf2, promoting its activation (orange pathway). Once activated, Nrf2 translocates to the nucleus, where it regulates ARE-driven cytoprotective genes that mitigate infection-induced ROS. In parallel, PERK–eIF2α–ATF4 signaling reprograms macrophage metabolism and inflammatory responses, promoting M1-like activation, mitochondrial adaptation, and enhanced defense against *S. aureus*. Solid arrows indicate positive regulation or activation. Blunt-ended lines indicate inhibitory or suppressive effects. Dashed arrows indicate proposed relationships. A question mark indicates the unknown relationship. An asterisk indicates the *in vitro* binding of CDC-48 and SKI-II.

ROS exert a dual role during infection, functioning as both essential antimicrobial effectors and potential mediators of host damage (72). In macrophages, NOX2-dependent oxidative burst promotes pathogen clearance (73), whereas excessive ROS generated from host and bacterial stress can cause oxidative damage (73), making the balance between antimicrobial activity and host protection a key determinant of infection outcome. In this study, we present evidence that SKI-II regulates SKN-1 activity in *C. elegans* as well as the orthologous Nrf2 pathway in mammalian cells (Fig. 7, upper part) and observed apparent beneficial effects of reduced ROS in both *C. elegans* (Fig. S1B) and macrophages (Fig. 3D). However, the readouts of these beneficial effects differ. In nematodes, intestinal bacterial load increased with SKI-II treatment, which reflects improved physiological activity as severely infected worms often enter a “sleep-like” state and reduce pharyngeal pumping (43). In macrophages, SKI-II treatment reduced intracellular bacterial load. This enhanced antimicrobial activity may be attributed to SKI-II-induced macrophage M1 polarization, which promotes pro-inflammatory cytokine secretion and activates antimicrobial effector pathways (74). One piece of evidence is that a key antimicrobial effector Lcn2, which sequesters bacterial siderophores, is the third most upregulated gene following SKI-II treatment in RAW264.7 cells based on our RNA-seq data. Therefore, these findings support a unified, context-dependent model in which SKI-II improves host resilience, manifesting primarily as tolerance in whole-animal systems and as a combination of resistance and protection in immune cells.

Although CDC-48 and VCP are structural and functional homologs, their relationships with SKN-1/Nrf2 signaling differ in important ways though characterizing them as acting in strictly opposite directions is an oversimplification. In the mammalian system, VCP negatively regulates Nrf2 by extracting ubiquitylated Nrf2 from the KEAP1-CUL3 complex for proteasomal degradation, and accordingly, the SKI-II inhibition on VCP triggers the downstream Nrf2 pathway activation (Fig. 7). However, in *C. elegans*, the SKN-1 activation may not be due to the dysfunction of CDC-48. Previously published work in *C. elegans* demonstrated that CDC-48 is required for SKN-1 activation during infection, and that loss of CDC-48 induces ER stress while suppressing SKN-1 signaling in *C. elegans* (50). Importantly, CDC-48 loss does not suppress SKN-1 directly but triggers the ER unfolded protein response (UPR^ER), which, in turn, inhibits SKN-1C through mutual inhibition between these two stress pathways (50). It also showed that CDC-48 knockdown would not affect the SKN-1 nuclear localization, consistent with our findings shown in Fig. S3A. Moreover, our data revealed no changes in the basal GFP level of the *gst-4* reporter under *cdc-48* RNAi (Fig. 2C through E). These observations indicate that CDC-48 is neither a positive nor a negative regulator of basal SKN-1 activity but rather is required for SKI-II-induced SKN-1 activation through a mechanism that remains to be fully elucidated. These results suggest that although SKI-II can bind CDC-48, its role in activating SKN-1 may not be achieved through inhibition of CDC-48. Additionally, SKI-II did not induce ER stress or attenuate established ER stress in worms (Fig. S3B and C), suggesting that SKI-II does not significantly suppress or enhance CDC-48-mediated ER homeostasis. One observation is that *C. elegans* RNA-seq data comparing the transcriptional profiles with and without SKI-II treatment revealed that SKI-II primarily induced xenobiotic stress-response pathways rather than canonical ER stress pathways (Fig. 2F). Therefore, although CDC-48 in *C. elegans* and its mammalian homolog VCP share highly conserved structural and functional roles in maintaining protein homeostasis, their regulatory relationships with the SKN-1/Nrf2 signaling pathways appear to be distinct, as robust ER stress pathway activation is observed in RAW264.7 macrophages but not in *C. elegans* (Fig. 5A; Fig. S3B). In *C. elegans*, the ER stress response and SKN-1 pathway share the same upstream regulator IRE-1 (50). This integrated regulatory network requires a balanced interaction between ER stress signaling and SKN-1-mediated stress adaptation. In contrast, mammalian cells possess multiple parallel signaling branches that regulate ER stress responses and Nrf2 signaling (75, 76), which do not rely on a single shared upstream regulator. Instead, ER stress and Nrf2 activation can be differentially regulated depending on the cellular context. Overall, the differential regulation of SKN-1/Nrf2 signaling between *C. elegans* and mammalian cells may reflect evolutionary divergence in stress adaptation strategies. As a small free-living nematode directly exposed to environmental toxins and microbes, *C. elegans* relies heavily on rapid xenobiotic detoxification and stress-response programs for survival. In contrast, mammalian systems possess specialized organs and multicellular buffering mechanisms that can initially accommodate chemical stress through protein quality control, metabolic adaptation, and immune regulation before mounting broader detoxification responses.

In macrophage RAW264.7 cells, we demonstrated that SKI-II binds to ATPase domains of VCP to inhibit its activity (Fig. 4B and F). The inhibition of VCP may modulate Nrf2 levels through two distinct mechanisms: impairing Nrf2 extraction from the KEAP1–CUL3 complex to prevent degradation (20), and inducing ER stress that activates PERK, which directly phosphorylates Nrf2 (77, 78). As pharmacological inhibition of PERK with GSK2606414 abolished SKI-II–induced Nrf2 activation (Fig. 5F and G). It suggests that although VCP inhibition may contribute to Nrf2 stabilization, SKI-II-induced Nrf2 activation in RAW264.7 macrophages is predominantly mediated through PERK signaling. Therefore, our data support the close connection between PERK and Nrf2. However, whether PERK directly phosphorylates Nrf2 in macrophages remains to be explored.

There are other connections between PERK and Nrf2. As described in Fig. 5C, PERK can also affect Nrf2 via p62/SQSTM1(64), and Nrf2 subsequently transcriptionally upregulates p62, reinforcing a positive feedback loop (79) (Fig. 7, lower part). Our RNA-seq data in mouse macrophages revealed a significant upregulation of SQSTM1 (Fig. 5B). Interestingly, SYing et al. found that *S. aureus* can interfere with Nrf2 signaling by disrupting the non-canonical p62/SQSTM1–Keap1 complex, thereby inhibiting Nrf2 activation (80). Thus, in combination with published data, we suggest that SKI-II may restore Nrf2 activity by promoting SQSTM1-mediated Keap1 degradation during *S. aureus* infection.

Relevant to SKI-II-mediated effects on Nrf2, Nicolas et al. reported that SKI-II induces the formation of inactive dimerized KEAP1 (32), thereby facilitating the release of Nrf2 to activate downstream gene transcription although the precise mechanism and direct molecular targets of SKI-II were unclear. Our findings suggest a possible mechanism by which inactive KEAP1 dimers may be generated through PERK-dependent Nrf2 activation. Specifically, it has been reported that the PERK effector ATF4 can bind to the COX-2 promoter and regulate its expression (81). COX-2 activity leads to the production of prostaglandins, including 15-deoxy-Δ¹²,¹⁴-prostaglandin J₂ (15d-PGJ₂), an endogenous electrophilic lipid capable of covalently modifying the cysteine-rich protein KEAP1 (82, 83). And COX-2 was significantly upregulated in RAW264.7 macrophages following SKI-II treatment. Combining the literature and our data, we propose that SKI-II inhibits VCP and activates PERK signaling, leading to ATF4-dependent induction of COX-2 (Fig. 7, lower part). The resulting increase in 15d-PGJ₂ may then disrupt the KEAP1–Nrf2 complex by covalently modifying KEAP1, thereby releasing Nrf2 to drive antioxidant gene expression (Fig. 7, lower part). The detailed mechanism remains to be investigated and verified.

Beyond its role in oxidative stress regulation, our study identifies SKI-II as a potent inducer of macrophage M1 polarization, an activity that has not been previously characterized. Mechanistically, our data suggest that this pro-inflammatory reprogramming is mediated, at least in part, through the activation of the PERK signaling pathway. It should be noted that the relationship between PERK and macrophage polarization is context-dependent while PERK activation has been shown to promote M1-like polarization under certain ER stress conditions (67, 84). Other studies have demonstrated that PERK signaling is critical for immunosuppressive M2 macrophage function, promoting mitochondrial respiration and lipid oxidation (84). The pro-inflammatory M1 polarization observed in our system may therefore reflect the specific context of SKI-II-induced VCP inhibition and ER stress in RAW264.7 macrophages. ATF4 has been shown to directly regulate inflammatory gene expression by binding to the COX-2 and IL-6 promoters (85, 86), thereby regulating their transcription. Although there is no direct evidence that ATF4 binds to the TNF-α promoter, previous studies have demonstrated a critical role for ATF4 in TNF-α activation (87). These observations, together with our findings, support a model in which ATF4 functions as a central transcriptional node coordinating cytokine and inflammatory mediator expression in SKI-II-treated macrophages, revealing a previously unexplored role for PERK–ATF4 signaling in this context.

Interestingly, a body of work has shown that ROS modify VCP through oxidation and S-glutathionylation of the critical residue Cys-522, inhibiting its ATPase activity and altering interactions with ER-associated degradation (ERAD) cofactors (88). Integrating this redox control of VCP into the ER stress, PERK autophosphorylation, and Nrf2 activation model, we propose a regulatory pathway for Nrf2 activation by which host cells sense and respond to bacterial challenge (Fig. 7, lower part). In this model, pathogen attack elevates ROS, which impairs VCP, triggering ER stress and PERK activation, and then promotes Nrf2 signaling both by PERK-dependent phosphorylation of Nrf2 and by inducing p62- and COX-2-mediated KEAP1 degradation, thereby stabilizing Nrf2 and enhancing its nuclear translocation. Nrf2 subsequently engages AREs to drive cytoprotective transcriptional programs that quell oxidative stress. In parallel, PERK–eIF2α–ATF4 signaling reshapes macrophage inflammatory and metabolic programs, supporting M1-like polarization and a shift in mitochondrial respiration that enhances antimicrobial ability and decreases ROS production (Fig. 7, lower part). Importantly, this VCP-associated pathway leading to PERK activation and Nrf2 induction may function independently of IRG-1/itaconate signaling, a pathway known to mediate Nrf2 activation in response to LPS in macrophages (89). Our RNA-seq data show that LPS markedly increases Acod1 expression (log_2_ fold change = 9.8) in RAW264.7 macrophages, whereas SKI-II reduces Acod1 transcription (log₂ fold change = −3.5), indicating that the antioxidant effects observed under these conditions are not driven by enhanced IRG-1 activity. In other words, SKI-II modulates oxidative stress through an IRG-1/itaconate-independent mechanism. Collectively, our study establishes a novel host-directed paradigm in which pharmacological modulation of VCP-associated, PERK-dependent Nrf2 activation reprograms macrophage metabolism and function, paving the way for therapeutic approaches that enhance innate immunity against bacterial infection.

## MATERIALS AND METHODS

Bacterial strains, cell lines, and additional experimental details used in this study are provided in the supplemental material.

### Immunomodulator screen

Approximately 50 young adult worms expressing the indicated GFP reporter were transferred into each well of a 384-well plate, with a volume of 30 µL per well. To each well, 35 µL of 20% TSB medium in M9 buffer was added. Five microliters of the diluted compounds was added to achieve a final concentration of 7.143 µg/mL. The kinetics of GFP intensity were then measured at 2-h intervals for 24 h using a Cytation 3 multimode reader (BioTek, USA). DMSO was used as a negative control.

We used two approaches to identify hits. First, the *z*-score was calculated: *z*-score = (*X* − μ)/σ, where *X* is the raw score, μ is the mean of the population, and σ is the standard deviation (SD) of the population. In our case, *X* is the raw GFP intensity value. The mean and SD were calculated using values of the whole plate. Second, all GFP intensity values were normalized to the value from the first time point, and then the *z*-score was calculated. If the *z*-score was higher than two for more than half of the time points either using the absolute or normalized GFP, the compound was deemed to be a hit on the primary screen. Primary hits were verified using the same protocol as the primary screen. The GFP value from 8 wells of DMSO blank controls was used to calculate the mean and SD in *z*-score. Verified hits had *z*-scores higher than 2 for over half of the time points.

#### *C. elegans*-MRSA liquid infection assay

*C. elegans-*MRSA liquid-based infection assays were performed essentially as previously reported (6). Synchronized young adult AU37 [*glp-4(bn2);sek-1(km4)*] *C. elegans* were infected with *S. aureus* MW2 (final OD_600_ of 0.04) in 96-well half-area plates containing 10% TSB in M9 buffer and treated with serially diluted compounds. After incubation at 25°C, worms were washed and stained with Sytox Orange to identify dead animals. The fluorescence images were acquired using a Cytation 3 multimode reader. Worm survival was calculated as the percentage of non-stained worms relative to the total number of worms. Vancomycin was used as a positive control. At least six wells per condition and three independent biological replicates were analyzed.

#### ROS measurement

ROS measurement in *C. elegans*: For ROS measurement in the liquid-based *S. aureus* infection assay, *C. elegans* were incubated in 10% TSB medium containing *S. aureus* MW2 at an OD₆₀₀ of 0.04 (100 worms per well in a 96-well half-area plate). Worms were treated with DMSO (negative control), 16 μg/mL vancomycin (positive control), or 16 μg/mL SKI-II and incubated under infection conditions for 3 days. After incubation, worms were washed nine times with M9 buffer using a Microplate Washer (BioTek, USA). Fifty micromolar of CM-H_2_DCFDA was then added to each well to achieve a final concentration of 25 μM CM-H_2_DCFDA (90). The ROS level was determined by measuring fluorescence kinetics at 469–525 nm using the Cytation 3 multimode reader (BioTek, USA).

ROS measurement in murine macrophages: RAW264.7 macrophage cells were seeded in a 96-well plate until reaching 95% confluency. Cells were challenged by *S. aureus* MW2 at an MOI of 10 for 2 h and were gently washed twice with 100 μL of pre-warmed DPBS. CM-H_2_DCFDA was added to the cells at a final concentration of 2.5 μM, along with SKI-II at a final concentration of 4 μg/mL. The ROS level was measured by fluorescence kinetics at 469–525 nm using the Cytation 3 multimode reader (BioTek, USA).

#### Intercellular bacterial burden assay

RAW264.7 macrophages were cultured in DMEM supplemented with 10% FBS and 1% penicillin–streptomycin at 37°C in a humidified 5% CO_2_ incubator and seeded into 96-well plates at 5 × 10⁴ cells per well. After 16 h of attachment, cells were infected with exponential-phase *S. aureus* MW2 at an MOI of 10 for 1 h. Following infection, macrophages were washed twice with pre-warmed DPBS and incubated in 100 µg/mL gentamicin for 2 h to eliminate extracellular bacteria. Cells were then washed twice with pre-warmed DPBS and incubated in medium containing 25 µg/mL gentamicin together with DMSO control, 4 µg/mL vancomycin, or 4 µg/mL SKI-II for 24 h. The following day, macrophages were washed twice with pre-warmed DPBS and lysed with 50 µL of 0.5% Triton X-100 in PBS. Lysates were serially diluted in PBS and plated onto TSB agar plates for CFU enumeration the next day.

#### AlphaLISA and ELISA assay

RAW264.7 macrophages were cultured in DMEM supplemented with 10% FBS and 1% penicillin-streptomycin at 37°C until they reached ~80% confluence. Cultures were washed with serum-free DMEM and incubated for 4 or 16 h under the following conditions: untreated control (serum-free DMEM), vehicle control (0.04% DMSO), positive control thapsigargin (0.3 µg/mL), or SKI-II (4 µg/mL). For PERK phosphorylation inhibition, cells were pretreated with the PERK inhibitor GSK2606414 (1 µM) for 1 h, followed by co-incubation with vehicle control (0.04% DMSO), the positive control thapsigargin (0.3 µg/mL), or SKI-II (4 µg/mL).

For the AlphaLISA assay measuring PERK phosphorylation (AlphaLISA SureFire Ultra p-PERK assay kit, Revvity): cells were lysed in 80 µL of 1× lysis buffer for 10 min at room temperature with shaking, and 10 µL of lysate was transferred to a 384-well OptiPlate. AlphaLISA detection was performed according to the manufacturer’s two-plate protocol: 5 µL acceptor mix was added and incubated for 1 h at room temperature, followed by 5 µL donor mix under subdued light and an additional 1 h incubation in the dark. Plates were read on a Spark multimode plate reader (Tecan, Switzerland) using AlphaLISA settings (excitation 680 nm, emission 615 nm), and signal intensities were normalized to protein concentration measured using BCA in parallel wells.

For the ELISA assay measuring Nrf2 levels (Mouse Nrf2 ELISA Kit, Biomatik): cells were washed with cold PBS twice and lysed in 100 µL of T-PER Tissue Protein Extraction Reagent (Thermo Scientific, USA) with 1× protease inhibitor cocktail (Fisher Scientific, USA). Cells were pipetted up and down several times and incubated on ice for 15 min. The lysates were pipetted repeatedly, incubated on ice for 15 min, and then clarified by centrifugation at 10,000 rpm for 10 min at 4°C. Nrf2 concentrations were quantified according to the manufacturer’s instructions (Biomatik, Canada) and normalized to total protein, as measured by the BCA assay in parallel wells.

#### RNA-seq

##### Macrophage RAW264.7

Compound treatment and total RNA extraction from RAW264.7 cells were performed as described for the qPCR assay. Cells were treated with 4 μg/mL SKI-II or an equivalent amount of DMSO as the control, followed by total RNA extraction. The extracted RNA was sequenced by Azenta Life Sciences (Burlington, MA, USA).

##### *C. elegans* AU37

Approximately 3,000 young adult AU37 worms were incubated in 10% TSB medium containing either DMSO (control) or 16 µg/mL SKI-II in a 6-well plate. Worms were maintained at 25°C for 8 h. After incubation, worms were collected into 1.5 mL microcentrifuge tubes and washed twice with M9 buffer by centrifugation at 800 × *g* for 1 min. The worm pellet was resuspended in 400 µL of lysis buffer from the Tissue Total RNA Isolation Kit V2 (Vazyme). Subsequently, 300 µL of 1.0 mm zirconia beads (BioSpec Products) was added, and samples were vortexed for 1 min to disrupt the worms. The lysates were processed according to the manufacturer’s protocol, including genomic DNA removal, RNA purification, washing, and elution steps. Purified RNA samples were submitted to AZENTA Life Sciences (Burlington, MA, USA) for RNA sequencing.

Raw data of sequence reads were trimmed to remove possible adapter sequences and nucleotides with poor quality using Trimmomatic v.0.36. The trimmed reads were mapped to the reference genomes using the STAR aligner v.2.5.2b. After the extraction of gene hit counts, the gene hit counts table was used for downstream differential expression analysis. Using DESeq2, a comparison of gene expression between the groups of samples was performed. The Wald test was used to generate *P-*values and log2 fold changes. Genes with adjusted *P*-values <0.05 and absolute log_2_ fold changes >1 were called as differentially expressed genes for each comparison.

### Statistics

Statistical analyses were performed using GraphPad Prism 10. Data are presented as mean ± SEM unless otherwise stated. Comparisons among multiple groups were analyzed using one-way ANOVA followed by Tukey’s or Dunnett’s multiple-comparison test, and comparisons between two groups were analyzed using unpaired two-tailed Student’s *t*-tests. Area under the curve (AUC) values were calculated for time-course fluorescence or ROS measurements. Sample sizes (*n*) are indicated in figure legends, and all experiments were independently repeated at least three times. Statistical significance was defined as NS: *P* > 0.05; *: *P* < 0.05; **: *P* < 0.01; ***: *P* < 0.001.

## Data Availability

RNA-seq count and normalized expression data have been deposited in the NCBI Gene Expression Omnibus (GEO) under accession number GSE310254. The rest of the data generated or analyzed during this study are all included in the published article and its supplemental material. All other data are available from the corresponding authors upon reasonable request.

## SUPPLEMENTAL MATERIAL

Supplemental material mbio.01558-26-s0001.pdf Fig. S1 to S6 and additional experimental details.

ASM does not own the copyrights to Supplemental Material that may be linked to, or accessed through, an article. The authors have granted ASM a non-exclusive, world-wide license to publish the Supplemental Material files. Please contact the corresponding author directly for reuse.
