# Supplementary material for: Identification of SKI-II as a host-protective immunomodulator against Staphylococcus aureus infection
Source: mBio. 2026 Aug 10;17(9):e01558-26. doi: 10.1128/mbio.01558-26 (PMC13556259; doi:10.1128/mbio.01558-26)
Supplement: Supplementary file 1 [file mbio.01558-26-s0001.pdf]

Supporting Information for

Identification of SKI-II as a host-protective immunomodulator against *Staphylococcus aureus* infection

Liyang Zhang[1], Kai Ye[2], Charilaos Dellis[1], Lewis Oscar Felix[1], Narchonai Ganesan[1], Biswajit Mishra[1,3], Aijun Zhang[1,3], Dale J Hamilton[1,3], Yizhi Jane Tao[2], and Eleftherios Mylonakis[1,3*]

*Corresponding authors: Eleftherios Mylonakis

Email: emylonakis@houstonmethodist.org

1Department of Medicine, Houston Methodist Hospital, Houston, TX 77030, USA

2Department of BioSciences, Rice University, Houston, TX 77005, USA

3Department of Medicine, Weill Cornell Medical College, New York, NY 10021, USA

Supplemental Figure 1

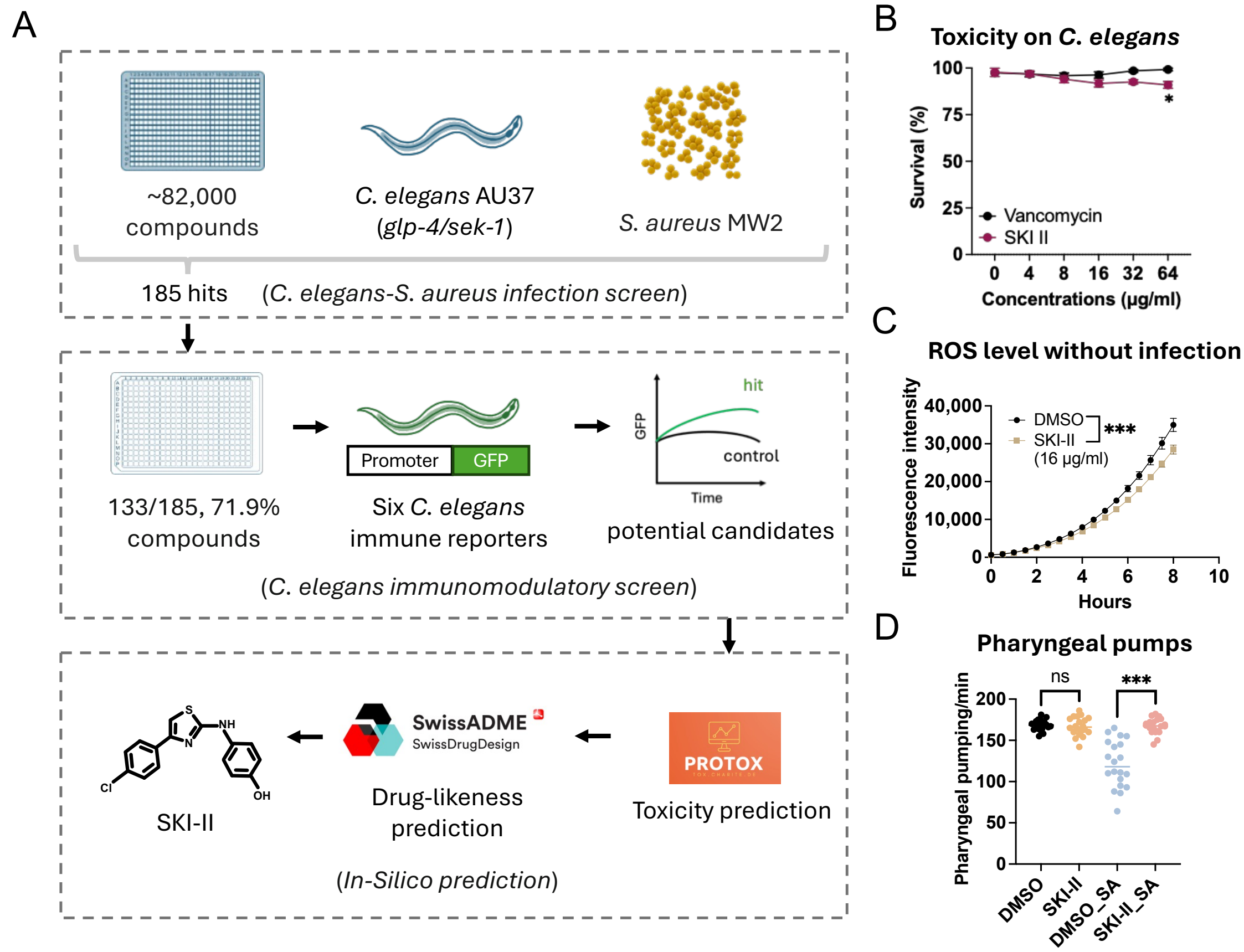

Figure S1. Identification of SKI-II as an immunomodulator in a *C. elegans*-based screen

(A) Schematic representation of the experimental procedure used to identify compounds with immunomodulatory potential. The screen for identifying immunomodulators was modified from the original infection screen by removing MRSA and keeping everything else the same, such as 10% TSB in M9 buffer and a concentration of 7.14 µg/ml for each compound.

(B) Toxicity assessment of SKI-II on *C. elegans* AU37. Worm survival was calculated after 5 days of incubation without infection. Statistical analysis was performed using the Kruskal–Wallis test followed by Dunn's multiple-comparison test, with each SKI-II treatment group compared to the untreated control (n = 8 biological replicates). *: $p < 0.05$.

(C) ROS levels in *C. elegans* AU37 worms without *S. aureus* MW2 infection following treatment with DMSO (control) or 16 µg/ml of SKI-II. The ROS level was obtained from a pool of ~50 worms in liquid. Area Under the Curve (AUC) values were calculated for each replicate, and statistical comparisons were performed using an unpaired t-test; ***: $p < 0.001$.

(D) Pharyngeal pumps of *C. elegans* AU37 under DMSO (control) or 16 µg/ml of SKI-II with or without *S. aureus* (SA) infection. The pharyngeal pumps were counted after 24 hours of infection. Each group has 20 worms. Statistical comparisons were performed using one-way ANOVA with Dunnett's multiple comparisons test. ***: $p < 0.001$.

Supplemental Figure 2

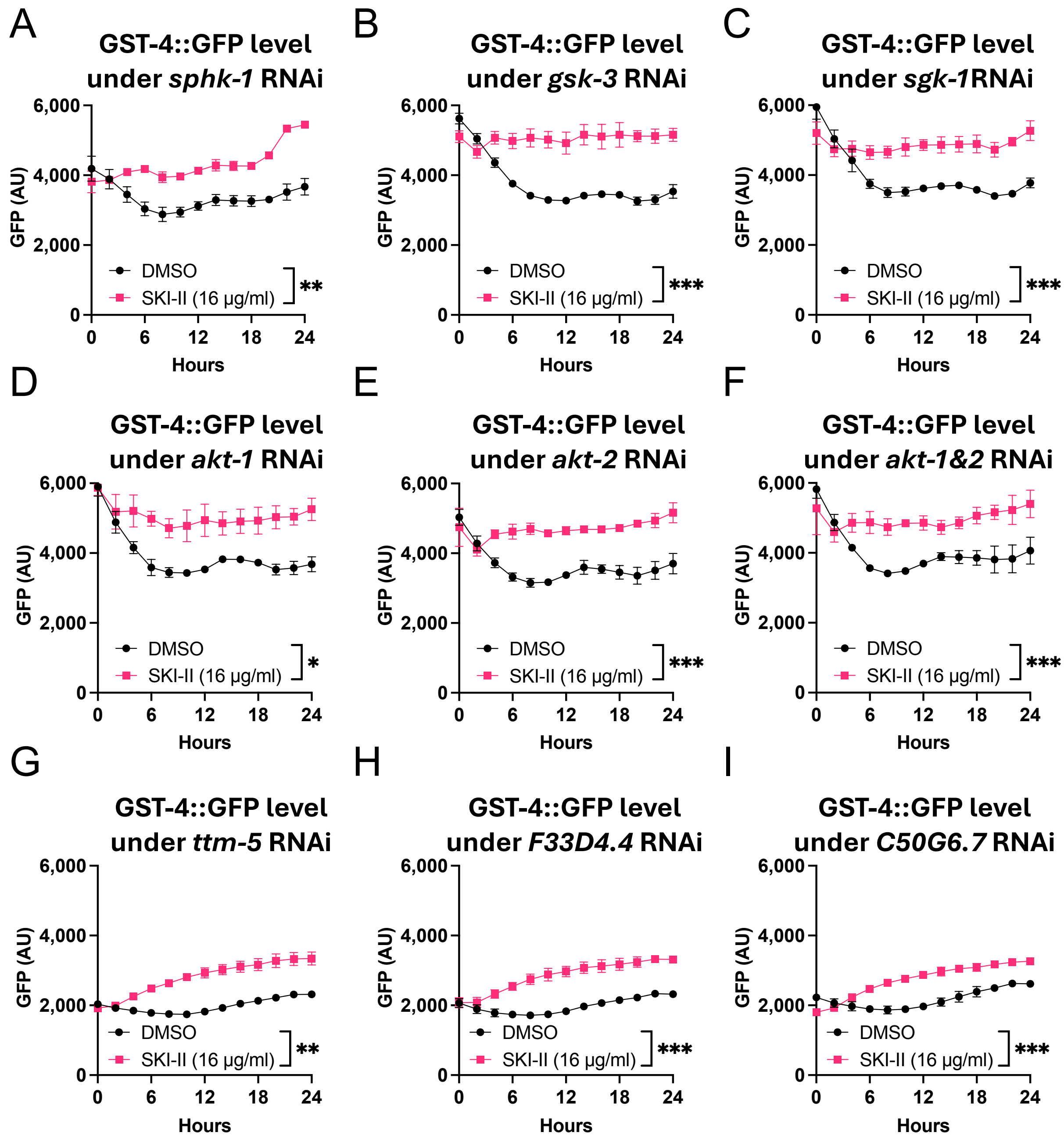

Figure S2. SKI-II targets the SKN-1/Nrf2 pathway

(A–I) GFP expression in *gst-4p::gfp* worms treated with DMSO or SKI-II (16 µg/ml) over time under RNAi knockdown of the following genes: (A) *sphk-1*, (B) *gsk-3*, (C) *sgk-1*, (D) *akt-1*, (E) *akt-2*, (F) *akt-1* & *akt-2* (double RNAi), (G) *ttm-5,* (H) *F33D4.4*, and (I) *C50G6.7*. Area Under the Curve (AUC) values were calculated from time-course GFP intensity for each biological replicate, and data are shown as mean ± SEM (n = 3, number of independent experiments). Statistical comparisons between DMSO and SKI-II treatments were performed using unpaired t-tests. NS: $p > 0.05$; *: $p < 0.05$; **: $p < 0.01$; ***: $p < 0.001$.

Supplemental Figure 3

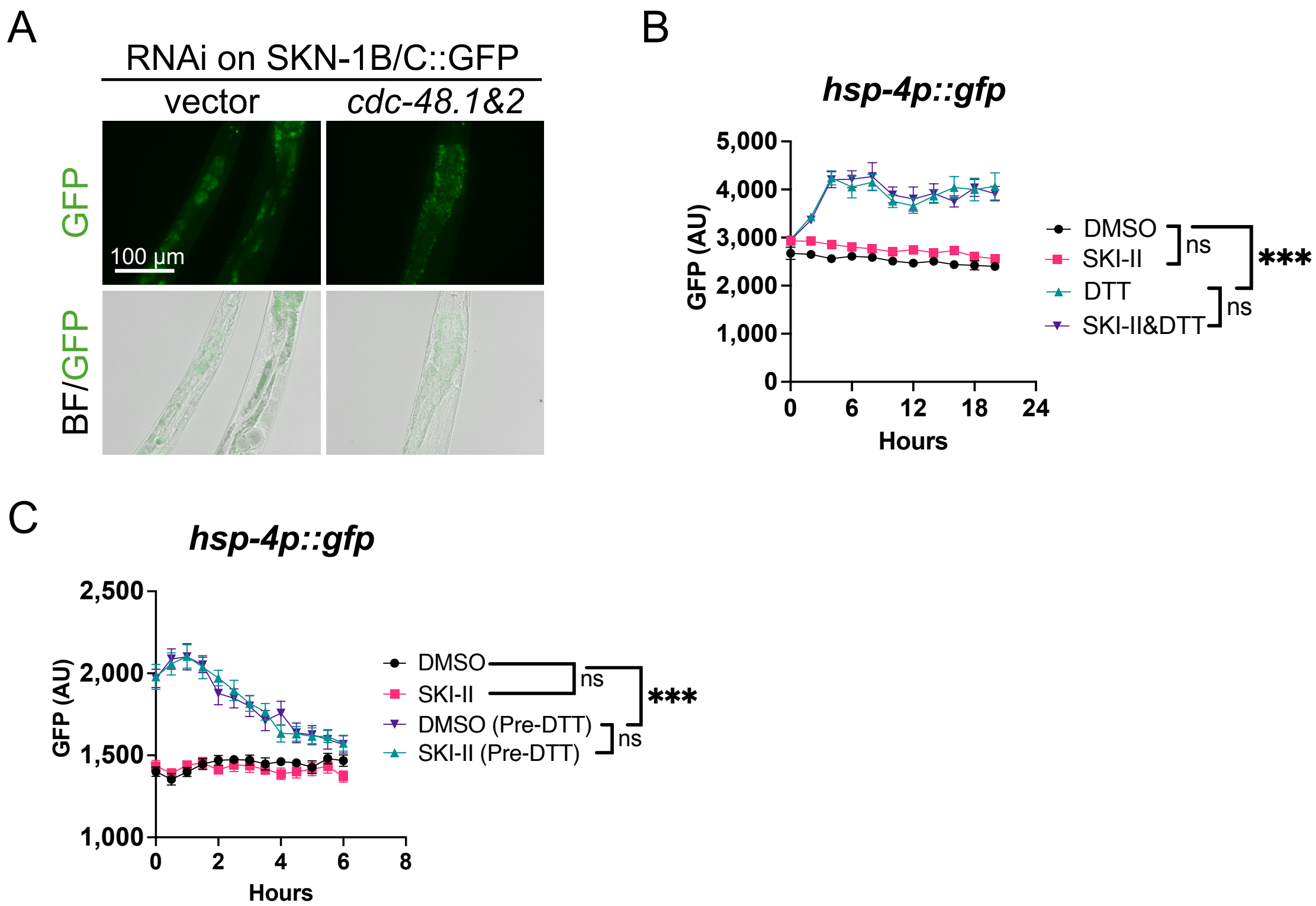

Figure S3. SKI-II neither inhibits nor enhances CDC-48 function to mediate SKN-1 activation. (A) Representative images of SKN-1B/C::GFP worms under empty vector control and cdc-48.1/2 RNAi treatment. GFP fluorescence images are shown in the upper panels, and bright-field/GFP merged images are shown in the lower panels. (B, C) GFP expression in ER stress indicator *hsp-4p::gfp* (B) or DTT-pretreated *hsp-4p::gfp* (C) incubated with DMSO, SKI-II (16 µg/ml), DTT (3 µg/ml), or SKI-II (16 µg/ml) & DTT (3 µg/ml). Area Under the Curve (AUC) values were calculated from time-course GFP intensity for each biological replicate, and data are shown as mean ± SEM. Statistical comparisons were performed using one-way ANOVA followed by Dunnett's multiple comparison test for panel (n = 6, number of biological replicates). ns: $p > 0.05$; ***: $p < 0.001$.

Supplemental Figure 4

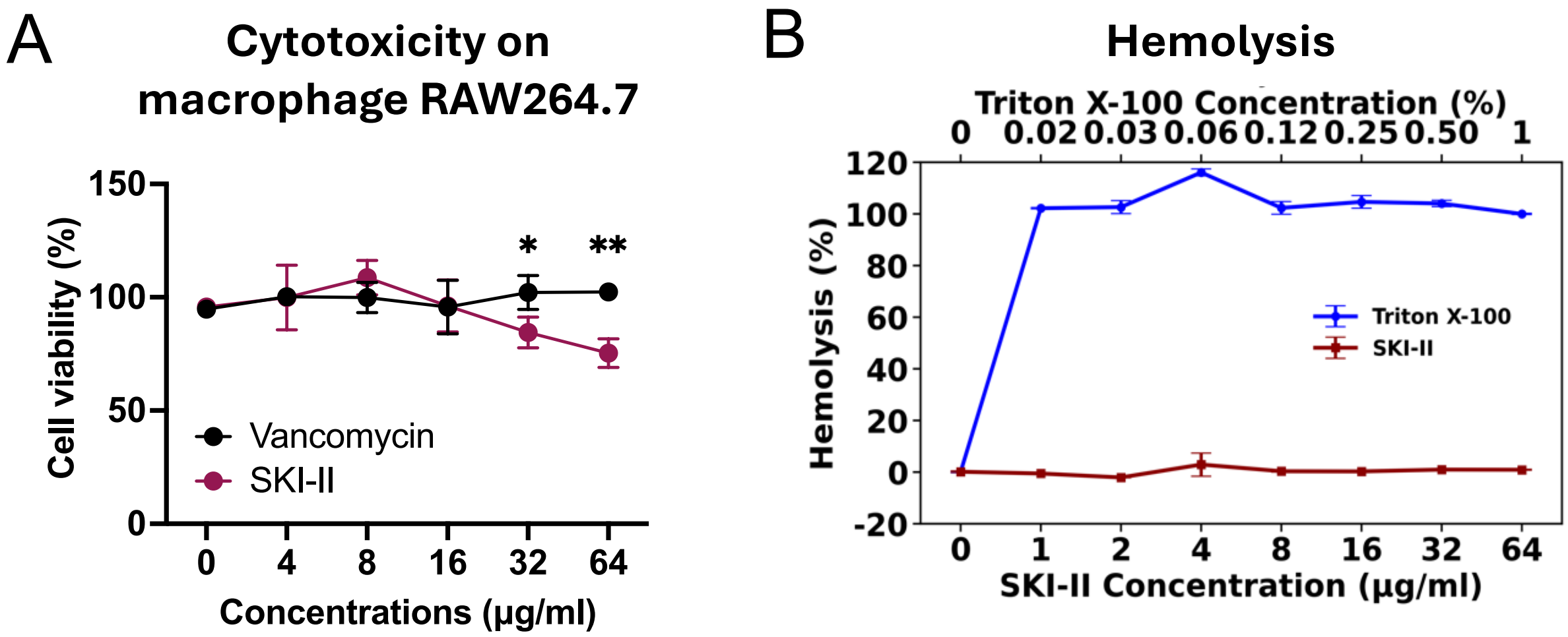

Figure S4. SKI-II exhibits low cytotoxicity

(A) Cell viability of RAW 264.7 macrophages after 24 h treatment with varying concentrations of DMSO, vancomycin, or SKI-II. Viability was normalized to untreated cells (100%). Statistical analysis (n = 3, number of independent experiments) comparing SKI-II treatment with untreated control was performed using one-way ANOVA with Dunnett's multiple comparisons test. *: $p < 0.05$; **: $p < 0.01$.

(B) Hemolytic activity of SKI-II compared to Triton X-100 (positive control). Hemolysis (%) was calculated relative to 1% Triton X-100 as 100% lysis.

Supplemental Figure 5

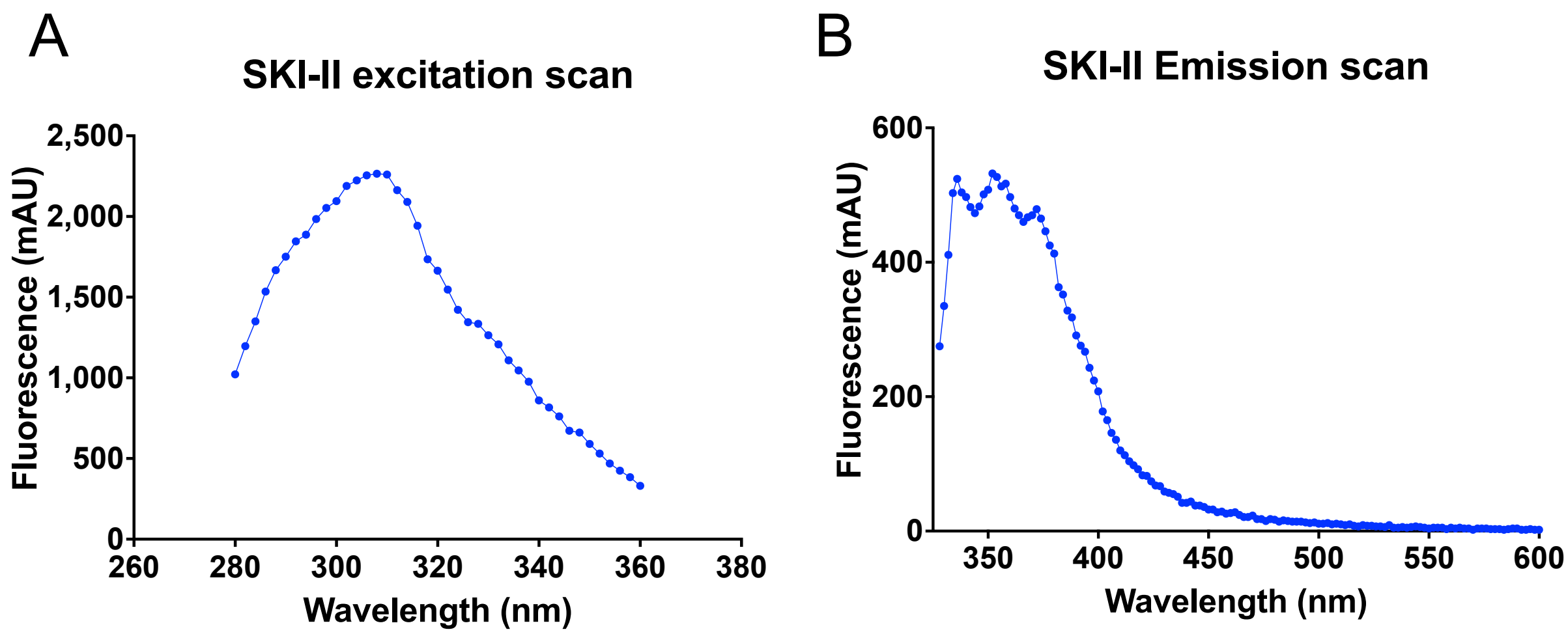

Figure S5. Fluorescence excitation and emission scan of SKI-II.

Since SKI-II contains a few phenol groups, we suspect this compound has an UV excitation and emission spectrum. Multiple iterations of scans were performed on a Tecan Spark plate reader and resulted in final excitation (A) and emission (B) spectra. (A) The excitation spectrum of SKI-II was examined with a gain of 100 at an emission wavelength of 390 nm with a bandwidth of 10 nm, the longest wavelength shoulder of the major emission peak with a range of 280 to 360 nm, a bandwidth of 5 nm, and a step of 2 nm. The maximum fluorescence was observed at 308 nm. (B) The emission spectrum of SKI-II was then determined with a gain of 100 and an excitation wavelength of 308 nm with a bandwidth of 5 nm. To avoid crosstalk between excitation and emission signals, the emission spectrum was examined from 328 to 600 nm with a bandwidth of 5, and a step of 2 nm. The peaks of fluorescence that can be used in the fluorescence polarization assay can be found at 352 nm and 372 nm.

Supplemental Figure 6

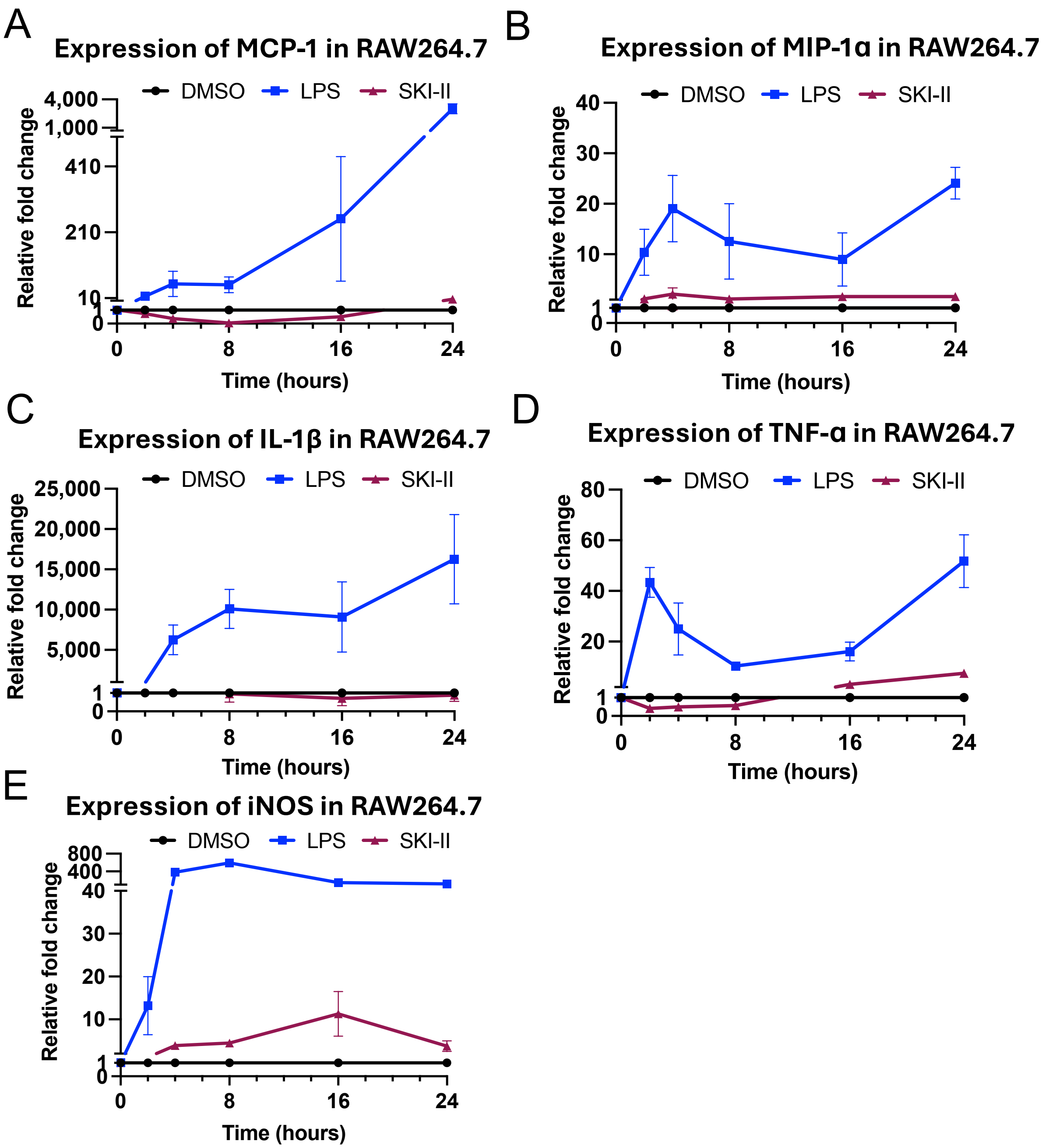

Figure S6. SKI-II induces pro-inflammatory responses in macrophages.

(A–E) Time-course expression of pro-inflammatory markers MCP-1 (A), MIP-1 (B), IL-1β (C), TNF-α (D), and iNOS (E) in RAW 264.7 macrophages treated with DMSO control,

SKI-II (4 µg/mL), or LPS (4 µg/mL) for 0, 8, 16, and 24. Cytokine levels were measured by qRT-PCR, and normalized to DMSO controls.

Supplemental materials and methods

Bacterial strains and cell lines

Escherichia coli strains OP50 and HB101 were used as food for *C. elegans* growth, *E. coli* HT115(DE3) carrying RNAi-expressing plasmids were used for RNAi-mediated gene knockdown in *C. elegans,* and *E. coli* BL21(DE3) was used for protein expression and purification. *Staphylococcus aureus* MRSA (MW2), *Acinetobacter baumannii* (ATCC 19606), *Pseudomonas aeruginosa* (PA14), *Enterobacter aerogenes* (EAE2625), and *Klebsiella pneumoniae* (H17185). were used for infection and MIC assays. The murine macrophage cell line RAW264.7 was used for innate immunity studies.

C. elegans *strains*

The AU37 strain (*glp-4(bn2); sek-1(km4) mutant*) with enhanced sensitivity to pathogens and temperature-sensitive sterility was used for *S. aureus* killing assays. AU37 was reared at 15°C and maintained at 25°C when it was used in immunity-related assays. Innate immunity reporter strains *gst-4p::gfp, nlp-29p::gfp, irg-1p::gfp, dod-24p::gfp, clec-60p::gfp, irg-5p::gfp* were used in screens identifying putative immunomodulators. *skn-1B/C::gfp* was obtained from Dr. D. Garsin, University of Texas Health Center at Houston

Plasmid constructs and protein purification

pQE9-His-p97(wt) was a gift from Graham Warren (Addgene plasmid # 14666; http://n2t.net/addgene:14666; RRID:Addgene_14666)(1). *C. elegans* CDC-48.1 and -48.2 coding sequences were purchased from Genscript and cloned into a pETDuet-His vector to generate an N-terminal His tag as His-p97. His-p97, His-CDC-48.1, and His-CDC-48.2 were purified following the protocol described by Meyer et al.(1).

Fluorescence polarization assay

Excitation and emission spectra were recorded for 10 nM SKI-II at room temperature on a Spark® Multimode Microplate Reader (Tecan, Switzerland) using low-volume 384-well black/clear flat-bottom polystyrene NBS microplates (Corning, USA). After fine-tuning the wavelengths, SKI-II showed an excitation maximum at 308 nm with emission maxima at 352 and 372 nm. Fluorescence polarization (FP) assays were then performed with hVCP and CDC-48 in FP buffer (20 mM MOPS, pH 7.2, 200 mM NaCl, 10 mM $MgCl_2$). Reactions (25 µL total) contained 100 nM SKI-II (from 5 µL of a 500 nM stock) and increasing concentrations of protein (20 µL per well) using excitation and emission wavelength of 308 nm and 352 nm respectively, with a bandwidth of 20 nm and a gain of 100%. Parallel and perpendicular fluorescence intensities were recorded and anisotropy was calculated; binding curves were fit by non-linear regression (GraphPad Prism, USA) to estimate K_D. All experiments were performed in triplicate, and data are reported as mean values.

To determine the mode of VCP ATPase activity inhibition by SKI-II, FP assays were performed with additional ATP in the reactions. 10 nM SKI-II was incubated with 500 nM protein which saturates FP measurements. A final concentration of 0, 10, or 50 nM of ATP was added to the reaction which were then mixed by pipetting up and down. FP values were immediately taken after mixing. Fraction saturation of 5 replicates was extrapolated from standard curves generated from the binding curve fitted by non-linear regression with the highest R^2 value.

Antimicrobial susceptibility assay

Minimal Inhibitory Concentrations (MICs) were determined using the broth microdilution method in accordance with the guidelines established by the Clinical and Laboratory

Standards Institute (CLSI)(2). Briefly, overnight bacterial cultures were diluted in cation-adjusted Mueller-Hinton Broth (caMHB) to a final concentration of approximately 1×10^6 CFU/mL. For each assay, 50 µL of the diluted bacterial suspension was added to individual wells of a sterile 96-well microtiter plate (Corning, NY, USA) containing 50 µL of test compounds pre-diluted in caMHB at 2× the intended final concentrations. Untreated bacterial suspensions served as negative controls, while wells containing only caMHB functioned as blanks to correct for background absorbance. The plates were incubated at 37°C for 18 hours. Following incubation, bacterial growth was assessed by measuring optical density at 600 nm (OD_{600}) using a SpectraMAX 250 microplate reader (Molecular Devices, CA, USA). An OD_{600} value of ≥0.05 was considered indicative of bacterial growth. All MIC determinations were performed in technical triplicates and repeated in at least two independent biological experiments to ensure reproducibility.

C. elegans *toxicity assay*

A *C. elegans* cytotoxicity assay was developed based on the *C. elegans*-MRSA infection assay. Briefly, approximately 20 young adult AU37 [*glp-4*(*bn2*);*sek-1*(*km4*)] worms in 35 µl M9 and diluted compounds with desired concentrations in 35 µl M9 buffer were added to each well of a clear-bottom 96-well plate. The plates were covered with an air-permeable membrane and incubated at 25°C for five days. Then, worms were washed 2 times with M9 buffer and were stained with 50 µL of 1 µM Sytox® Orange solution (Life Technologies, USA) per well for 16 hours. Excess stain was washed away three times with M9 buffer and then fluorescence and bright-field micrographs were captured with a Cytation 3 multimode reader (BioTek, USA). Only dead worms were stained by Sytox, whereas live worms remained unstained. Worm survival was calculated as the ratio of non-

Sytox-stained worms to the total worms per well. Compound toxicity was determined by comparing the survival rate of treated worms to that of the untreated control group. At least six wells for each condition were analyzed per replicate, and three independent biological replicates were performed.

skn-1B/C::gfp *expression assay*

Approximately 3,000 synchronized L1-stage *C. elegans* were placed onto NGM plates seeded with an *E. coli* OP50 lawn and incubated at 25°C. After 48–52 hours, worms were washed six times with 40 mL of M9 buffer containing 0.01% Tween-20. Following washing, 50 worms were transferred into each well of a 384-well plate in a total volume of 15 µL per well. 35 µL of 20% TSB medium in M9 buffer and 20 µL of SKI-II (final concentration: 16 µg/mL) were added to each well. DMSO was used as a negative control. Worms were then incubated at 25°C for 24 hours, and images were captured using a BZ-X800 fluorescence microscope (Keyence, Japan) with either a 4X or 100X objective lens. Both the brightfield and GFP fluorescence channels were used for imaging. The worms exhibiting strong and weak GFP signals were manually counted. At least 300 worms were counted for each biological replicate and three replicates were performed. For CDC-48 RNAi, L2-stage *skn-1B/C::gfp* worms were dropped on the CDC-48.1&2 RNAi plate and incubated till young adult (24-36 hours at 25 °C). Worms were washed with M9 buffer and were imaged as described above.

RAW264.7 cell toxicity assay

RAW264.7 macrophages were seeded in a 96-well plate at 10,000 cells per well in DMEM medium containing 10% FBS and 1% penicillin-streptomycin. After 40 hours of incubation at 37°C in a 5% CO_2 incubator, the cell medium was aspirated, and fresh medium

containing the desired compound concentrations was added to each well at 100 μl per well. The cells were treated with the compound for 24 hours. Subsequently, 70 μl of XTT working solution (2,3-bis-(2-methoxy-4-nitro-5-sulfophenyl)-2H-tetrazolium-5-carboxanilide) was directly added to each well. One row without cells was used as a negative control. The plate was then incubated at 37°C for 4 hours. After incubation, absorbance was measured at 450 nm and 660 nm using the Cytation 3 multimode reader (BioTek, USA), where the 450 nm reading reflects XTT-specific absorbance and the 660 nm reading accounts for background signals from cell debris or other non-specific sources. The specific absorbance of the sample was calculated using the formula: Specific Absorbance = [Abs450 nm(Test) – Abs450 nm(Blank)] – Abs660 nm(Test). The specific absorbance signal from the treated cells was compared to that of the dilution series or untreated cells to assess the number of viable cells per well. Three biological replicates were performed.

Quantitative PCR

RAW264.7 macrophage cells (2.5×10^5 per well) were seeded in a 24-well plate with DMEM containing 10% FBS and 1% penicillin-streptomycin. The plate was incubated at 37°C with 5% CO_2 for 20 hours. After incubation, the cell medium was aspirated, and fresh medium containing the compound at the desired concentrations was added to each well (500 μL per well). Following incubation for the designated time, cells were washed twice with 1 mL DPBS and then directly lysed in the 24-well plate. Genomic DNA was removed, and total RNA was extracted using a commercial RNA purification kit (Tissue total RNA isolation kit, RC112-01, Vazyme, China). RNA concentration was measured using a NanoDrop spectrophotometer. qPCR was performed using a CFX-96 real-time

thermocycler (Bio-Rad, USA) with Taq Pro Universal SYBR qPCR Master Mix (Vazyme, China). Gene expression levels were normalized to β-Actin, and relative expression was analyzed using the *ΔΔ*Ct method. Primer sequences used for qPCR(3) (5′→3′; F = Forward, R = Reverse):

TNF-α F: tgtctcagcctcttctcattcct, R: ggtttgctacgacgtgggctacag;

IL-1β F: ccttttgacagtgatgagaatgac, R: ccacgggaaagacacaggtag;

IL-6 F: ttcacaagtccggacaggag, R: tggtcttggtccttagccac;

MCP-1 F: ctcacctgctgctactcattc, R: gcttgaggtggttgtggaaaa;

MIP-1α F: acaagcagcagcgagtacca, R: catgatgttgagcaggtgacaga;

iNOS F: gcctcatgccattgagttcatcaacc, R: gagctgtgaattccagagcctgaag;

COX-2 F: cagcaaatccttgctgttcc, R: tgggcaaagaatgcaaacatc;

β-Actin F: cctggctggccgggacctgac, R: accgctcgttgccaatagtgatga.

Three biological replicates were performed for each condition.

Seahorse extracellular flux assay

Mitochondrial function was assessed using the Seahorse XF Cell Mito Stress Test Kit (Agilent Technologies, USA) according to the manufacturer's protocol. RAW264.7 macrophages were seeded at 8×10^4 cells per well in XF96 cell culture microplates and incubated for 16 hours at 37°C in a humidified incubator with 5% CO_2 to allow adherence. The next day, cells were incubated with DMSO control or SKI-II (4 µg/ml) for two hours. One hour prior to the assay, cells were washed and incubated with Seahorse XF Assay Medium supplemented with 25 mM glucose, 1 mM pyruvate, and 2 mM glutamine, and placed in a non-CO_2 incubator at 37 °C for equilibration. Sequential injections of mitochondrial modulators were performed to evaluate respiratory parameters: Oligomycin

(1.5 μM) to inhibit ATP synthase (complex V) and measure ATP-linked respiration; FCCP (1 μM) to uncouple mitochondrial respiration and determine maximal respiration and spare respiratory capacity; and rotenone (0.5 μM) and antimycin A (0.5 μM) to inhibit complex I and III, respectively, and assess non-mitochondrial respiration. Oxygen consumption rate (OCR) and extracellular acidification rate (ECAR) were measured using the Seahorse XFe96 Analyzer. Data were analyzed using Wave software (Agilent Technologies, USA).

ER stress indicator assay

Approximately 50 young adult worms expressing the indicated GFP reporter were transferred into each well of a 384-well plate, with a volume of 30 μl per well. To each well, 35 μl of 20% TSB medium in M9 buffer was added. The kinetics of GFP intensity were then measured at 2-hour intervals for 24 hours using a Cytation 3 multimode reader (BioTek, USA). DMSO was used as a negative control. DTT (3 μg/ml) was used as a positive control of ER stress. The concentration of SKI-II was 16 μg/ml. For the DTT pretreatment assay, worms were first incubated with 3 μg/ml of DTT for 2 hours, then DTT was washed off with M9 buffer. Worms were incubated with DSMO or SKI-II as described above.

Worm pharyngeal pumping assay

S. aureus infection was performed using the established *C. elegans*–MRSA liquid infection assay. After 24 h of infection, worms were transferred onto NGM plates, and pharyngeal pumping was assessed under a stereomicroscope. The number of pharyngeal pumps was counted for 1 min for each worm.

References

1. Meyer HH, Shorter JG, Seemann J, Pappin D, Warren G. 2000. A complex of mammalian ufd1 and npl4 links the AAA-ATPase, p97, to ubiquitin and nuclear transport pathways. EMBO J 19:2181-92.
2. Sader HS, Flamm RK, Jones RN. 2013. Antimicrobial activity of daptomycin tested against Gram-positive pathogens collected in Europe, Latin America, and selected countries in the Asia-Pacific Region (2011). Diagn Microbiol Infect Dis 75:417-22.
3. Mao X, Pan X, Peng X, Cheng T, Zhang X. 2012. Inhibition of titanium particle-induced inflammation by the proteasome inhibitor bortezomib in murine macrophage-like RAW 264.7 cells. Inflammation 35:1411-8.
